# Sandwich‐Structured Fluorinated Polyimide Aerogel/Paraffin Phase‐Change Composites Simultaneously Enables Gradient Thermal Protection and Electromagnetic Wave Transmission

**DOI:** 10.1002/advs.202411758

**Published:** 2024-12-06

**Authors:** Tao Shi, Jianwei Jing, Zhiqiang Qian, Gaojie Wu, Guofeng Tian, Huan Liu, Xiaodong Wang

**Affiliations:** ^1^ State Key Laboratory of Organic–Inorganic Composites Beijing University of Chemical Technology Beijing 100029 China; ^2^ State Key Laboratory of Polymer Materials Engineering College of Polymer Science and Engineering Sichuan University Chengdu Sichuan 610065 China; ^3^ Key Laboratory of Green and High‐End Utilization of Salt Lake Resources Qinghai Institute of Salt Lakes Chinese Academy of Sciences Qinghai Provincial Key Laboratory of Resources and Chemistry of Salt Lakes Xining Qinghai 810008 China; ^4^ Key Laboratory of Carbon Fiber and Functional Polymers (The Ministry of Education) Beijing University of Chemical Technology Beijing 100029 China

**Keywords:** fluorinated polyimide aerogel, heat insulation, low dielectric constant, phase change materials, sandwich‐structured composites

## Abstract

There is an emerging requirement of advanced functional materials for simultaneous thermal protection and electromagnetic wave‐transparent transmission applications. A novel polyimide (PI) aerogel‐based sandwich‐structural composite is developed to meet such a requirement in this study. This composite is based on a unidirectional fluorinated PI (FPI) aerogel as a lower layer, a nondirectional conventional PI aerogel as a middle layer, and a nondirectional FPI aerogel/paraffin phase‐change composite as an upper layer. The lower layer exhibits a unique unidirectional porous microstructure and an ultralow dielectric constant of 1.04. The upper layer possesses a dynamical temperature regulation capability thanks to its loaded paraffin having a high latent heat capacity of 242.7 J g^−1^. The presence of the nondirectional PI aerogel middle layer can effectively prevent against the leakage of paraffin from the upper layer to the surface of the composite. Through a rational integration of three functional layers, the developed sandwich‐structured composite not only can provide gradient thermal protection for hot objects over a long period but also exhibits an excellent wave‐transparent capability to establish communication between two electromagnetically shielded electronic devices. With such prominent thermal insulation and wave‐transparent functions, the sandwich‐structured composite exhibits great potential for specific applications in aircraft, spacecraft, radar systems, and satellite communication.

## Graphical abstract

A novel type of fluorinated polyimide aerogel‐based sandwich‐structural composite is developed for simultaneous applications in gradient thermal protection and electromagnetic wave‐transparent transmission. Through the innovative design and rational integration of three different functional layers, the developed sandwich‐structured composite exhibits great potential for specific applications in aircraft, spacecraft, radar systems, and satellite communication.

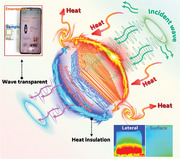

## 1 Introduction

Aerogels, as a class of porous and lightweight functional nanomaterials, have attracted great attention in academia and industry, and their pore and skeletal structure can be regulated to accomplish superior properties, such as large specific surface area, low density, high porosity, and low thermal conductivity.^[^ ^1^ ^]^ For example, the design of anisotropic hierarchical structures in aerogels using a directional freezing technology can significantly reduce the heat transfer in the through‐thickness direction of the anisotropic aerogels, providing a better thermal insulation capability than their isotropic counterparts.^[^ ^2^ ^]^ With these outstanding properties, aerogels have gained diverse applications in industrial and civil areas, including microwave absorption, energy storage, and dielectrics.^[^ ^3^ ^]^ In particular, aerogels have been widely used in the temperature regulation and thermal management of buildings, cryogenics, subsea systems, space, and civil engineering due to their superior thermal insulation ability.^[^ ^4^ ^]^ The pore walls of aerogels can reduce heat radiation, while the air contained in aerogels can suppress convective heat transfer. Moreover, aerogel can contribute to a large specific surface area, which increases the heat conduction paths along the pore walls to limit solid heat conduction.^[^ ^5^ ^]^ To date, inorganic aerogels (e.g., SiO_2_, Al_2_O_3_, carbon nanotubes, MXene, and reduced graphene oxide)^[^ ^6^ ^]^ and organic aerogels [e.g., modified melamine, nanocellulose composite, chitosan, and polyimides (PIs)]^[^ ^7^ ^]^ have been developed for highly effective thermal insulation. Additionally, a highly porous structure enables aerogels to obtain a low dielectric constant for electromagnetic wave transmission. The thermal insulation and wave‐transparent characteristics of aerogels satisfy the essential requirements of radomes and antenna windows for electromagnetic communication of the internal electronic devices in aircraft and spacecraft.^[^ ^8^ ^]^ Some inorganic oxide and nitride ceramic aerogels with dielectric loss and constant and high thermal stability, such as SiO_2_, BN, and Si_3_N_4_, have been developed for synchronous wave‐transparent and heat‐resistant applications.^[^ ^9^ ^]^

Although inorganic aerogels exhibit superior frame retardancy and low dielectric characteristics, they have to suffer from poor mechanical performance. The skeletons of inorganic aerogels are generally connected by nanoparticles or nanosheets. This is the fundamental reason for their fragility, thus making it difficult to maintain structural integrity under compression or bending deformation.^[^ ^10^ ^]^ In contrast to inorganic aerogels, organic aerogels exhibit much better mechanical properties because of their flexible polymer network structure unquestionably beneficial to reinforce their compressive strength. Unfortunately, most organic aerogels present a flammable nature and poor thermal stability.^[^ ^11^ ^]^ Among the polymeric materials, PIs have drawn great attention due to their remarkable resistance to extreme conditions. The imide and aromatic heterocyclic rings in the main chains of PIs endow it to obtain excellent mechanical properties, high‐temperature resistance, and effective heat insulation. In this regard, various polyimide (PI)‐based composite aerogels^[^ ^12^ ^]^ and crosslinked PI aerogels^[^ ^13^ ^]^ have been developed for high efficient thermal protection. Furthermore, the dielectric constants of PIs can be reduced through molecular structure design, including the introduction of fluorine‐containing groups, alicyclic structures, and large‐volume rigid non‐planar conjugated structures into the molecular chains of PIs.^[^ ^14^ ^]^ Although the molecular and microstructure designs of PI aerogels have propelled the revolutionary development of satellite communication and radar systems, aerogels still have limitations in practical application under extreme conditions along with drastic temperature variations. In this circumstance, a large amount of heat generated from a high‐temperature object in a short time is hardly dissipated out of aerogels, leading to a failure risk for the internal electronic devices in aircraft and spacecraft.

In recent years, aerogel/phase change material (PCM) composites as a type of emerging heat energy‐storage composite material offer an effective solution for the short‐time overheating issue occurring in various application scenarios. PCMs are typical latent heat‐storage materials that can absorb and release huge amounts of heat energy through reversible phase transitions within a narrow temperature range. This enables the aerogel/PCM composites to maintain a low surface temperature through heat energy absorption by the PCM to reduce heat impact without consuming additional energy.^[^ ^15^ ^]^ During the heat energy‐absorption process, the aerogel skeletons can effectively prevent the flow and leakage of the PCM component in the molten state through capillary force or intermolecular interactions.^[^ ^16^ ^]^ The high porosity of aerogels can provide a high volume capacity to maximize the PCM loading, thus enabling the resultant phase‐change composites to gain a high latent‐heat capacity. In this sense, the aerogel/PCM composites have been proved to be a type of promising material for addressing the short‐time overheating issue through dynamic thermal regulation by their loaded PCMs.^[^ ^17^ ^]^ Nevertheless, the introduction of PCMs into aerogels also accompanies with a new hurdle. Owing to the limited heat absorption capacity of the loaded PCM, the aerogel/PCM composites usually present a gradually increasing trend in their surface temperature with the complete fusion of the PCM component. This cannot meet the requirement of long‐term use for thermal insulation under extreme temperature conditions. In addition, the dielectric constant of the aerogel/PCM composites is increased significantly due to the full filling of the aerogel pores by the PCM. In this case, there have been no public reports involved in the aerogel/PCM composites for the simultaneous application of thermal insulation and electromagnetic wave transmission. On the contrary, researchers have paid more attention to the development of PCM‐based composites for electromagnetic interference shielding and energy conversion, and these functionalized phase‐change composites include flexible multilayered phase‐change films,^[^ ^18^ ^]^ ZIF‐67/porous carbon‐encapsulated PCM,^[^ ^19^ ^]^ and MXene‐based phase‐change composites.^[^ ^20^ ^]^

**Figure 1:**
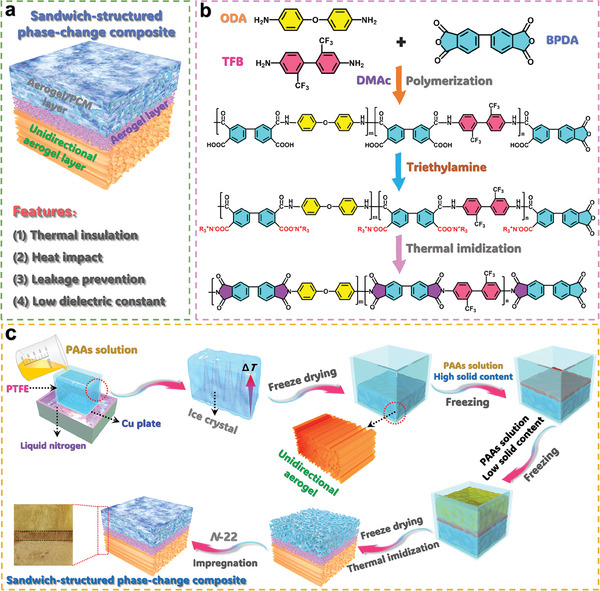
a) Schematic structure and features of sandwich‐structural composite. b) Reaction mechanism of FPI aerogel. c) Fabrication strategy of sandwich‐structured phase‐change composite.

In the present study, a hierarchical structuring strategy was proposed to construct a sandwich‐structural phase‐change composite system based on a fluorinated PI (FPI) aerogel as a supporting material and paraffin as an organic PCM for the simultaneous application of thermal protection and electromagnetic wave transmission. **Figure** **1**a depicts such a hierarchically structuring strategy, in which a sandwich‐structured composite was constructed with a unidirectional FPI aerogel as a lower layer, a high‐solid‐content nondirectional PI aerogel as a middle layer, and a nondirectional FPI aerogel/paraffin composite as an upper layer. With high porosity, good mechanical performance, low dielectric constant and dielectric loss, excellent flame retardance, and low thermal conductance, the lower layer can act as a barrier to insulate heat transfer to the surface of the resultant sandwich‐structured composite and as a passage to allow the electromagnetic wave to transmit into the electronic devices below the sandwich‐structured composite. *N*‐docosane (*N*‐22) was employed as a paraffin‐type PCM due to its high melting enthalpy of over 260 J g^−1^. Under extreme temperature conditions, such a high latent heat capacity enables the FPI aerogel/*N*‐22 composite upper layer to absorb huge amounts of thermal energy to buffer against the heat impact on the electronic devices in a short time. The C−H and C−C bonds in the *N*‐22 molecule can reduce the polarizability of the sandwich‐structural composite, weakening the negative influence on its dielectric performance based on the Debye equation.^[^ ^24^ ^]^ Moreover, the FPI aerogel matrix in the composite upper layer has the ability to prevent against the leakage of *N*‐22 in the molten state during its melting process. The PI aerogel middle layer achieved a partially closed pore structure due to its high solid content, which can further prevent against the exudation of the molten *N*‐22 from the upper layer to the lower one. Thanks to these characteristic compositions and unique microstructures of the unidirectional FPI aerogel, high‐solid‐content PI aerogel, and FPI aerogel/PCM composite as well as their rational combination, the sandwich‐structured composite developed in this work not only achieves a good thermal protection capability for long‐term thermal insulation, short‐term heat impact, but also presents low dielectric constant and loss.

The combination of aerogel and PCM‐infused aerogel has been reported by some researchers. For example, Li et al.^[^ ^21^ ^]^ reported a glass window consisting of silica aerogels and PCMs for thermal insulation in cold climates. Liu et al.^[^ ^22^ ^]^ reported an investigation on the superhydrophobic silica aerogels and their layer‐by‐layer structure for thermal management in harsh cold and hot environments. Liu et al.^[^ ^23^ ^]^ reported a Kevlar nanofibrous aerogel‐based 3‐layer tandem cloak for highly efficient and long‐lasting infrared stealth. These composites have a layer‐by‐layer structure through a combination of aerogels as a thermal insulation material and an organic PCM as a latent heat‐storage material, and their layers are independent and can be freely combined in practice. In this study, we designed an integral sandwich‐structure for the phase‐change composite by using a high‐density PI aerogel as a middle layer to connect the upper and lower layers. Such a connection was formed by chemical bonding through thermal imidization. Therefore, the resultant sandwich‐structured phase‐change composite can provide better structural stability and durability for the layer‐by‐layer composite system. Furthermore, the high‐solid‐content nondirectional PI aerogel as a middle layer can generate extra thermal insulation and thus enhance the total thermal protection effect of the sandwich‐structured composite. Based on innovative hierarchical integration of an FPI aerogel and its composite with the PCM, this study may offer valuable insights for the development of advanced functional materials and their structural designs aligned with the simultaneous application of thermal management and electromagnetic wave transmission.

## 2 Results and Discussion

### 2.1 Structural Characterizations of FPI Aerogels

**Figure 2:**
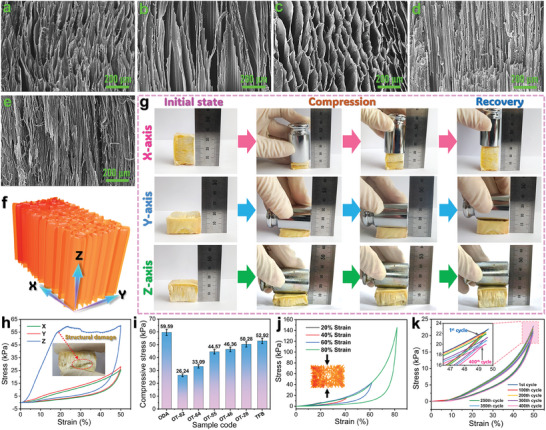
SEM images of a) ODA‐based, b) OT‐82‐based, c) OT‐55‐based, d) OT‐28‐based, and e) TFB‐based FPI aerogels. f) Schematic structure of unidirectional FPI aerogel. g) Digital photographs of OT‐64‐based FPI aerogel compressed in variable axial directions. h) Stress‐strain curves of OT‐64‐based FPI aerogel in different axial directions. i) Compressive stresses of unidirectional FPI aerogels. Stress‐strain curves of OT‐64‐based FPI aerogel at j) different ultimate strains and k) a specific ultimate strain with different cycle numbers.

In order to realize the simultaneous application of gradient thermal protection and electromagnetic wave transmission, a novel type of sandwich‐structured phase‐change composite system was designed with a unidirectional FPI aerogel as a lower layer, a high‐solid‐content nondirectional conventional PI aerogel as a middle layer, and a nondirectional FPI aerogel/paraffin composite as an upper layer (Figure 1a). In this composite system, the unidirectional FPI aerogel lower layer plays a key role in thermal insulation and thermal protection due to its characteristic fluorine‐containing chemical structure and unique unidirectional microstructure. To optimize the chemical compositions, microstructure, and thermal performance of the lower layer, a series of unidirectional FPI aerogels as a lower layer material were separately fabricated by directional freezing, freezing drying, and thermal imidization of fluorinated poly(amic acid) (PAA) precursors according to the fabrication strategy depicted in Figure 1b,c. The fluorinated PAA precursors were synthesized through polycondensation of *3,3′,4,4′*‐biphenyltetracarboxylic dianhydride (BPDA) with *4,4′*‐diaminodiphenyl ether (ODA) and *2,2′*‐bis (trifluoromethyl)benzidine (TFB) using *N*,*N*‐dimethylacetamide (DMAc) as a solvent (Figure S1, Supporting Information). To evaluate the influence of TFB on the thermal insulation and dielectric properties of the FPI aerogel, a series of unidirectional FPI aerogel samples with different TFB contents were prepared through controlling the molar ratio of ODA to TFB. The codenames of the as‐prepared unidirectional FPI aerogel samples and their composites with paraffin are listed in **Table** **1**. As seen in Figure S2 (Supporting Information), these unidirectional FPI aerogel samples exhibit a color change from golden to white with an increase in the TFB content. The scanning electron microscopy (SEM) images (**Figure** **2**a−e) show that these aerogel samples present a multilayer‐stacked microstructure in their transversal surfaces. Such a multilayer‐stacked structure can facilitate the heat transfer along the parallel direction of microchannels in the unidirectional FPI aerogels, thus generating a good thermal insulation effect on their perpendicular direction. There are some breakages observed from the unidirectional FPI aerogels at a high content of TFB. This may be ascribed to the existence of fluorine groups that confine the rotational freedom of the bonds in the backbone of the FPI molecular chains, resulting in a reduction in flexibility and an improvement in the rigidity accordingly. As a result of the introduction of fluorine groups, a higher TFB content leads to a brittle nature of FPI, and therefore there is a damaged microstructure generated during the preparation of unidirectional FPI aerogels as observed from their SEM image (Figure 2d).

**Table 1:** The sample codes and compositions of PI and FPI aerogels and their phase change composites.

Codename	Sample composition
ODA‐based PI aerogel	ODA + BPDA (unidirectional aerogel)
OT‐82‐based FPI aerogel	ODA (80 wt.%) + TFB (20 wt.%) + BPDA (unidirectional aerogel)
OT‐64‐based FPI aerogel	ODA (60 wt.%) + TFB (40 wt.%) + BPDA (unidirectional aerogel)
OT‐55‐based FPI aerogel	ODA (50 wt.%) + TFB (50 wt.%) + BPDA (unidirectional aerogel)
OT‐46‐based FPI aerogel	ODA (40 wt.%) + TFB (60 wt.%) + BPDA (unidirectional aerogel)
OT‐28‐based FPI aerogel	ODA (20 wt.%) + TFB (80 wt.%) + BPDA (unidirectional aerogel)
TFB‐based FPI aerogel	TFB + BPDA (unidirectional aerogel)
ODA@*N*‐22 composite	ODA‐based nondirectional PI aerogel loaded with *N*‐22
OT‐82@*N*‐22 composite	OT‐82‐based nondirectional FPI aerogel loaded with *N*‐22
OT‐64@*N*‐22 composite	OT‐64‐based nondirectional FPI aerogel loaded with *N*‐22
OT‐55@*N*‐22 composite	OT‐55‐based nondirectional FPI aerogel loaded with *N*‐22
OT‐46@*N*‐22 composite	OT‐46‐based nondirectional FPI aerogel loaded with *N*‐22
OT‐28@*N*‐22 composite	OT‐28‐based nondirectional FPI aerogel loaded with *N*‐22
TFB@*N*‐22 composite	TFB‐based nondirectional FPI aerogel loaded with *N*‐22
ODA/ODA@*N*‐22 composite	ODA‐based PI aerogel/high‐solid‐content ODA‐based nondirectional PI aerogel/ODA@*N*‐22 sandwich‐structured composite
OT‐46/OT‐46@*N*‐22 composite	OT‐46‐based FPI aerogel/high‐solid‐content ODA‐based nondirectional PI aerogel/OT‐46@*N*‐22 sandwich‐structured composite
TFB/TFB@*N*‐22 composite	TFB‐based FPI aerogel/high‐solid‐content ODA‐based nondirectional PI aerogel/TFB@*N*‐22 sandwich‐structured composite

As observed from the energy‐dispersive *X*‐ray (EDX) spectrum and corresponding elemental mapping images in Figure S3a (Supporting Information), there is a group of the C, N, and O elements in the ODA‐based PI aerogel along with a homogeneous elemental distribution. As for the OT‐55‐based and TFB‐based FPI aerogels, the F element was also found in their EDX spectra and elemental mapping images besides the C, N, and O elements (Figure S3b,c, Supporting Information). These elements can further be detected by the survey *X*‐ray photoelectron spectrometry (XPS) as seen in the XPS spectrum of the TFB‐based FPI aerogel (Figure S4a, Supporting Information). Its high‐resolution XPS core‐level spectra not only identify the imide rings (─CO─NH─ and C─N─C bonds) but also demonstrate the existence of the F─C bond as seen in Figures S4b–d and S5 (Supporting Information). These analysis results of the unidirectional FPI aerogels are in good agreement with their *Fourier*‐transform infrared (FTIR) spectra, in which the characteristic absorption peaks for the C─O, C=O, C─N, and C─F bonds can be observed at 1235, 1715, 1376, and 1192 cm^−1^, respectively (Figure S6, Supporting Information). The aforementioned characterization results demonstrate the successful fabrication of PI and FPI aerogels with the desired unidirectional microstructures and chemical compositions.

### 2.2 Mechanical Properties of Unidirectional FPI Aerogels

Figure 2f shows the schematic structure of the unidirectional FPI aerogels in three axial directions. The OT‐64‐based FPI aerogel as a representative sample can easily recover to its original shape after forcedly compressing in the *X*, *Y*, and *Z* directions (Figure 2g), suggesting good elasticity and flexibility. The degrees of compression in the *X* and *Y* directions are higher than that in the *Z* direction because of the anisotropic microstructure of the FPI aerogel. To quantify the mechanical properties of the unidirectional FPI aerogels, their stress‐strain behaviors were symmetrically characterized by using a universal material testing machine, and the resultant stress‐strain curves are presented in Figure 2h−k. The OT‐64‐based FPI aerogel as a representative sample exhibits a similar stress‐strain behavior in the *X* and *Y* directions but a different behavior in the *Z* direction as observed from its stress‐strain curves (Figure 2h). This confirms an anisotropy‐dependent mechanical property of the unidirectional FPI aerogels. The maximum stresses at a specific compressive strain of 50% were determined to be 25.6 and 28.0 kPa in the *X* and *Y* directions, respectively. In the *Z* direction, there is an abrupt stress‐growth region due to structural damage occurring in the multilayer‐stacked structure. It is worth noting that the stress‐strain curves of the unidirectional FPI aerogel present a narrower hysteresis loop only in the *X* and *Y* directions. This results in lower energy dissipation compared to that in the *Z* direction.

The effect of TFB content on the compressive stress of the unidirectional FPI aerogels was further investigated. All of the unidirectional FPI aerogels can recover completely at a specific compressive strain of 50% in the *X* direction, and their stresses present a growing trend with an increase in the TFB content (Figure S7, Supporting Information; Figure 2i). The introduction of the TFB moiety evidently increases the rigidity of the FPI molecular chain, enabling the TFB‐based FPI aerogel to obtain a compressive stress of 52.9 kPa. In contrast, the ODA‐based PI aerogel exhibits a higher compressive stress of 59.6 kPa than those of the FPI aerogels (Figure 2i). This may be attributed to the chain flexibility resulting from the incorporation of the ODA moiety. The flexible ODA segment can induce the accumulation and shrinkage of the PI molecular chains during the freeze‐drying and thermal imidization process, thus increasing the density and compressive stress of the ODA‐based PI aerogel. It should be noted that the density of the ODA‐based PI aerogel is fairly higher than that of the FPI aerogels (Figure S8, Supporting Information). Figure 2j shows the stress‐strain curves of the OT‐64‐based FPI aerogel at strains ranging from 20% to 80%. These stress‐strain curves were found to be with each other in *X*–direction at various strains during the compression process, indicating good resilience of the OT‐64‐based FPI aerogel. The OT‐64‐based FPI aerogel exhibits a significant increase in compressive stress with an increase in strain and achieves a maximum stress of 145.4 kPa at a specific strain of 80%. In addition, there is no significant reduction in the stress of the OT‐64‐based FPI aerogel after 400 compression‐decompression cycles, indicating that the unidirectional FPI aerogels possess superior long‐term stability in compressive resilience.

### 2.3 Hydrophobic and Dielectric Properties of Unidirectional FPI Aerogels

**Figure 3:**
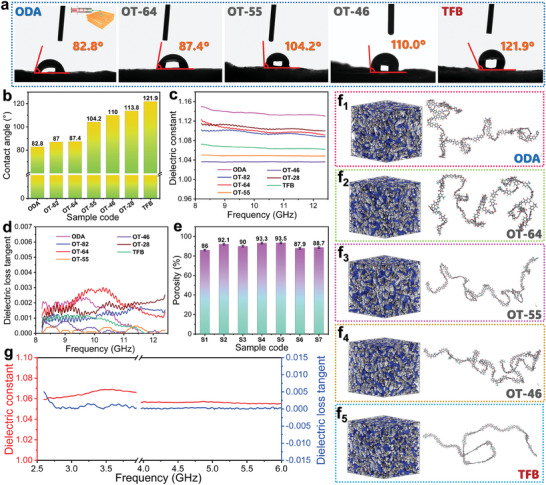
a) Water contact angle images and b) contact angle data of unidirectional FPI aerogels parallel to the channels. c) Dielectric constants and d) dielectric loss tangents of unidirectional FPI aerogels parallel to the channels (sample dimensions: 22.9 × 10.2 × 2.5 mm^3^). e) Porosities of S1) ODA‐based PI aerogel, S2) OT‐82‐based, S3) OT‐64‐based, S4) OT‐55‐based, S5) OT‐46‐based, S6) OT‐28‐based, and S7) TFB‐based FPI aerogels. f) Representative simulation models and steady states of PI and FPI molecules. g) Dielectric constant and loss tangents of OT‐46‐based FPI aerogel across the frequency range in 2.5−3.94 GHz (sample dimensions: 33.89 × 71.84 × 2.5 mm^3^) and 3.94−5.99 GHz (sample dimensions: 22.0 × 47.25 × 2.5 mm^3^).

The hydrophobic performance of the unidirectional and nondirectional FPI aerogels is evaluated by static water contact angle measurement, with the resultant results presented in **Figure** **3**a,b. The water contact angles of the unidirectional and nondirectional FPI aerogels were found to increase with an increase in the TFB content. This finding may be ascribed to the presence of the F atom on the FPI molecular chains, which not only reduces the polarizability of FPI but also increases their intermolecular distance and reduces their surface energy.^[^ ^25^ ^]^ It is noteworthy that the water contact angles of the unidirectional and nondirectional FPI aerogels are greater than 90° when their TFB contents are higher than 40%, indicating good hydrophobicity. The dielectric tests were conducted to evaluate the influence of the F atom and porous structure on the dielectric properties of the unidirectional FPI aerogels. Figure 3c,d shows the dielectric constants and loss tangents of the unidirectional FPI aerogels. All of the unidirectional FPI aerogels present low dielectric constants and loss tangents in the frequency range of 8.2–12.4 GHz. In particular, the dielectric constants of the unidirectional FPI aerogels are as low as 1.04–1.15, while their dielectric loss tangents are lower than 0.0035. Such low dielectric parameters are attributed to the multiporous structure of the unidirectional FPI aerogels as well as the introduction of TFB into the PI molecule chains. As shown in Figure 3e, these unidirectional FPI aerogels with different contents of TFB all show high porosity according to the results obtained from the mercury porosimetry tests (Figure S9 and Table S1, Supporting Information). High porosity can promote to a high content of air in the FPI aerogels, thus enabling them to gain a low dielectric constant due to the fact that the air has a dielectric constant close to 1.0. In contrast to the unidirectional FPI aerogels, the ODA‐based PI aerogel exhibits a slightly higher dielectric constant (1.14) and loss tangent (0.0014) due to its slightly smaller porosity, and however these two dielectric parameters are still much lower than those of conventional PI thin solid films.^[^ ^26^ ^]^

Compared to the ODA‐based PI aerogel, the FPI aerogels exhibit a lower dielectric constant (Figure 3c), and their porosities also increased with the introduction of TFB (Figure 3e). These results are in good agreement with the densities of the FPI aerogels (Figure S8, Supporting Information). The OT‐28‐based and TFB‐based FPI aerogels obtain a lower porosity than the other FPI aerogel samples. This may be due to the fact that a high content of TFB increases the rigidity of the FPI molecular chain to restrain the formation of a multiporous structure in its aerogels. However, the dielectric constant of the TFB‐based FPI aerogel is lower than those of the OT‐82‐based and OT‐64‐based FPI aerogels due to its higher TFB content. In addition to the porosity, the chemical structure also affects the dielectric behavior of PI aerogels. Figure 3f_1_−f_5_ and Figure S10 (Supporting Information) display the molecular simulation models and corresponding PI molecular chains simulated by using the Material Studio 8.0 software. The blue and gray areas in the models represent the free and occupied volumes, respectively. Details for the simulation procedure are presented in Section S2 (Supporting Information). According to the simulation results (Table S2, Supporting Information), a high content of TFB increases the fraction‐free volume of the FPI molecules, which reduces their dipole density significantly and also lowers their dipole moments. The low dipole moments and strong electron absorption effect of the F atom promote a decrease in the dielectric constants of FPI.^[^ ^27^ ^]^ Under the cooperative effects of high porosity and featured chemical structure, the OT‐46‐based FPI aerogel obtained the lowest dielectric constant among the unidirectional FPI aerogel samples. The OT‐46‐based FPI aerogel also exhibits low dielectric constants and loss tangents across the frequency range from 2.5 to 6.0 GHz (Figure 3g). These finds indicate that the unidirectional FPI aerogels have superior dielectric performance. This ensures they obtain a good wave‐transparent ability for electromagnetic communication. It should be noted that the FPI aerogels show an oscillatory behavior in their dielectric constants and dielectric tangents, which may be caused by a standard error of ≈0.001 on the vector network analyzer. In addition, the C─F bonds obtain the unique dipole relaxation kinetics, which can respond to an external magnetic field.^[^ ^28^ ^]^ Meanwhile, the fluoride atoms can induce the accumulation of charge carriers at the interface between the fluorine‐containing regions of the FPI matrix and pores, leading to the Maxwell‒Wagner polarization.^[^ ^29^ ^]^ As a result, both the dipole relaxation and Maxwell‒Wagner polarization caused the oscillatory behavior of the dielectric constant and dielectric tangent.

### 2.4 Thermal Insulation Performance of Unidirectional FPI Aerogels

**Figure 4:**
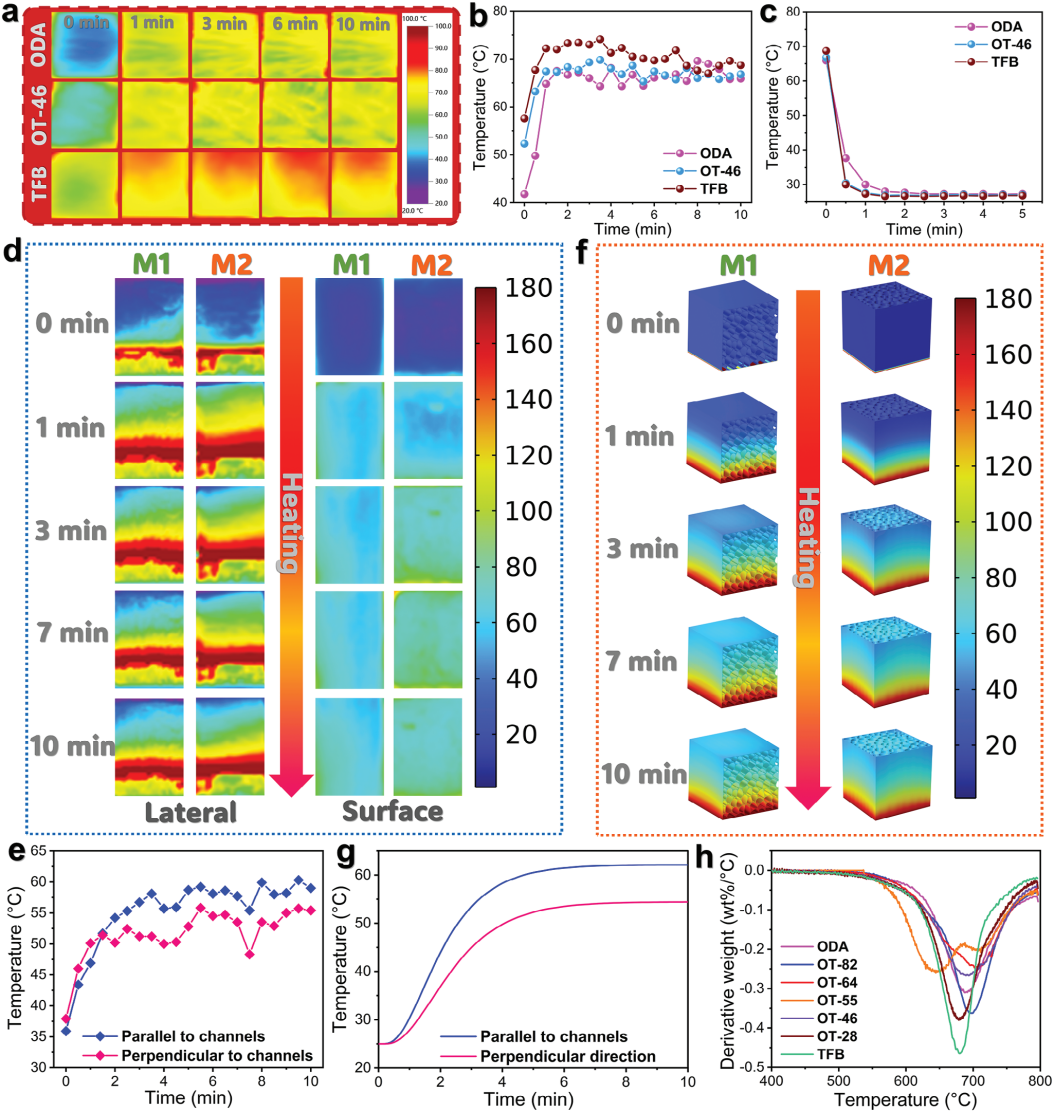
a) Infrared thermal images and b) corresponding temperature‐time evolution curves of ODA‐based PI aerogel and OT‐46‐based and TFB‐based FPI aerogels during heating at 180 °C. c) Temperature‐time evolution curves of ODA‐based PI aerogel and OT‐46‐based and TFB‐based FPI aerogels during natural cooling. d) Infrared thermal images and e) temperature‐time evolution curves of the laterals and surfaces of OT‐46‐based FPI aerogel (M1) perpendicular and (M2) parallel to the channels during heating at 180 °C. f) Simulated infrared thermal images and g) simulated temperature evolution‐time curves of aerogel parts (M1) perpendicular and (M2) parallel to the channels in the OT‐46‐based FPI aerogel at a heat source temperature of 180 °C. h) DTG thermograms of unidirectional FPI aerogels.

The thermal insulation properties of the unidirectional FPI aerogels were evaluated by using an infrared thermographic camera to record the thermal images of the ODA‐based PI aerogel and the OT‐46‐based and TFB‐based FPI aerogels as three representative samples during the heating process at 180 °C for 10 min, followed by natural cooling for 5 min. As seen in the infrared thermal images of three aerogel samples in the heating process (**Figure** **4**a), there is a rapid change in the colors of the three aerogel samples from blue to yellow, indicating a fast increase in their surface temperatures. It is notable that the surface temperatures of the three aerogel samples rise drastically within 1 min and then are maintained stably at ≈69 °C in the heating process (Figure 4b). This result may be ascribed to the existence of the air in the aerogel pores because the air can cause low thermal conductance for the aerogels to perform a convective heat transfer easily. As seen in Figure S11 (Supporting Information), most of the aerogel samples exhibit a thermal conductivity lower than 32 mW m^−1^ K^−1^. In addition, the loose skeleton and large specific surface area (Table S1, Supporting Information) of the aerogels can increase heat conduction paths to limit the solid heat conduction.^[^ ^30^ ^]^ As a result, the three aerogel samples exhibit a much lower surface temperature than the heat source (180 °C), indicating a good thermal insulation effect. To confirm the experimental reliability of the temperature recorded by the infrared thermographic camera, we further recorded the temperature‐time evolutions of the three representative samples by a *k*‐type thermocouple in the heating and cooling processes. The surface temperatures of the three aerogel samples are maintained stably at 70.7 °C for the ODA‐based PI aerogel, 68.4 °C for the OT‐46‐based FPI aerogel, and at 67.2 °C for the TFB‐based FPI aerogel (Figure S12, Supporting Information), which are very close to the results recorded by the infrared thermographic camera (Figure 4b). This result suggests that the temperature evolutions recorded by the infrared thermographic camera have good reliability. After natural cooling for 1 min, the three aerogel samples display a blue color on their surface (Figure S13, Supporting Information), and their surface temperatures decrease sharply within 0.5 min and then keep stable at ≈27 °C for a certain time (Figure 4c). This temperature evolution is also consistent with the results recorded by the thermocouple as shown in Figure S14 (Supporting Information). Such a fast temperature decrease is attributed to the low density of the PI and FPI aerogels, resulting in a low sensible heat storage capacity to store thermal energy.

To assess the influence of a unidirectional structure on the thermal insulation performance of the unidirectional FPI aerogels, infrared thermal images were recorded to obtain the temperature‐time evolution curves of the laterals and surfaces of the OT‐46‐based FPI aerogel perpendicular and parallel to the channels during the heating process. As seen in Figure 4d, the aerogel part parallel to the channels shows a slightly faster color change than that perpendicular to the channels, indicating a faster temperature change in the aerogel part parallel to the channels. The aerogel part parallel to the channels shows a higher surface temperature by 6.8 °C than that perpendicular to the channels (Figure 4e). Such a result is in good agreement with the temperature (5.7 °C) recorded by the *k*‐type thermocouple (Figure S15, Supporting Information). These results can be attributed to the unidirectional structure of the FPI aerogel. Such a unique unidirectional structure leads to a lower thermal conductivity of the aerogel part perpendicular to the channels (Figure S16, Supporting Information). In this case, the heat conduction in‐plane is prior to out‐of‐plane in the unidirectional FPI aerogels, delaying the heat transfer from the bottom to the top. This conclusion can further be verified by the heat transfer simulation. As seen in the simulated infrared thermal images (Figure 4f), the aerogel parts perpendicular and parallel to the channels show temperature evolutions similar to the experimental results. The surface temperature difference between the aerogel parts perpendicular and parallel to the channels was determined to be 7.7 °C, which is very close to the experimental result (Figure 4g). These findings confirm that the unidirectional structure of the FPI aerogels can generate an effective heat insulation effect and therefore facilitates their application in thermal protection. The thermal stability of the unidirectional FPI aerogels determines their service temperature for safety use, and it can be characterized by thermogravimetric analysis (TGA). As observed from the resultant TGA thermograms (Figure S17, Supporting Information), all of the unidirectional FPI aerogels were found to exhibit a one‐step thermal degradation behavior, and there is no significant mass loss found at a temperature of below 560 °C as shown in the differential thermogravimetric (DTG) thermograms (Figure 4h). This enables the unidirectional FPI aerogels to endure a temperature as high as 560 °C for heat insulation. The unidirectional FPI aerogels reveal a slight decrease in the characteristic temperature at a maximum degradation rate with an increase in the content of TFB. This may be ascribed to deterioration in the thermal stability of FPI at a high TFB content. In addition, there is a slight mass loss occurring at 150 °C due to the evaporation of residual solvents in the unidirectional FPI aerogels. Based on all of the aforementioned analysis results, the OT‐46‐based FPI aerogel was found to exhibit the optimal performance in dielectric behavior and thermal insulation among the as‐prepared FPI aerogel samples. Therefore, the OT‐46‐based FPI aerogel was determined to be the optimum lower‐layer material for the fabrication of the designed sandwich‐structural phase‐change composites.

### 2.5 Chemical Compositions and Thermal Properties of Phase‐Change Composites

**Figure 5:**
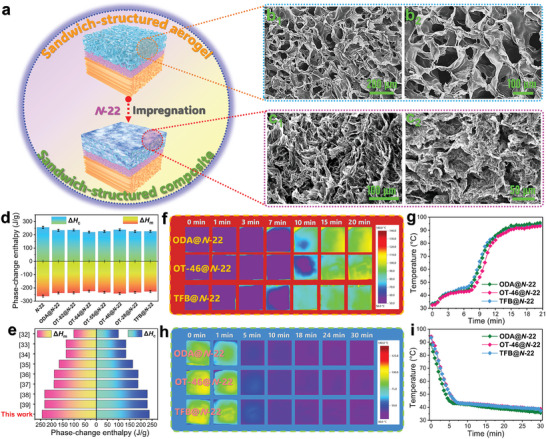
a) Fabrication strategy of sandwich‐structured phase‐change composites. SEM images of b) nondirectional OT‐46‐based FPI aerogel and c) OT‐46@*N*‐22 composite. d) Phase change enthalpies of pure *N*‐22 and its composites. e) Comparison of phase‐change enthalpies between the phase‐change composite developed in this work and paraffin‐based phase‐change composites reported in the literature. Infrared thermographic images and corresponding temperature‐time evolution curves of ODA@*N*‐22, OT‐46@*N*‐22, and TFB@*N*‐22 composites during the f,g) heating and h,i) cooling processes.

To construct the sandwich‐structured phase‐change composites designed by this study, a series of sandwich‐structured composite aerogels were first fabricated with a unidirectional FPI aerogel as the lower layer, a nondirectional PI aerogel obtained at a high solid content of PAA as the middle layer, and a nondirectional FPI aerogel as the upper layer (Figure 1). Then, *N*‐22 as a PCM was impregnated into the upper layer of the as‐prepared composite aerogels under a vacuum condition to form the desired sandwich‐structured composites (**Figure** **5**a). SEM and FTIR were performed to characterize the chemical compositions and thermal properties of the nondirectional FPI aerogel/*N*‐22 composites as the upper layer of the sandwich‐structured composites. As observed from the SEM images in Figure 5b_1_,b_2_, the nondirectional FPI aerogel layer exhibits a highly interconnected multiporous structure with a large pore size before being impregnated with *N*‐22. Such a large pore size provides a large internal space for the nondirectional FPI aerogel to load more amounts of *N*‐22 in their 3D multiporous cavity. After impregnation with *N*‐22, there is no void pore left in the aerogel layer and also no distinct interface between the nondirectional FPI aerogel and *N*‐22 as seen in Figure 5c_1_,c_2_. This finding suggests that the nondirectional FPI aerogel layer can offer intermolecular interaction and capillary force to engulfing *N*‐22 in their 3D cellular framework to prevent the fluidity and exudation of molten *N*‐22. Moreover, the FTIR spectrum of the OT‐46@*N*‐22 composite as a representative sample shows a series of characteristic absorption peaks for *N*‐22 (C─H bond: 728 and 2850 cm^−1^) and FPI (C=O bond: 1715 and 1781 cm^−1^; C─O bond: 1235 cm^−1^) (Figure S18, Supporting Information). These characterization results confirm the successful fabrication of the upper layer with the desired chemical compositions and microstructures for the sandwich‐structured composites.

The phase‐change behaviors and corresponding enthalpies of the nondirectional FPI aerogel/*N*‐22 composites were investigated by differential scanning calorimetry (DSC) to determine their latent heat‐storage capacity for future thermal management application of the sandwich‐structured phase‐change composites. Both pure *N*‐22 and its composite samples exhibit a similar bimodal phase‐transition behavior in the heating and cooling processes, in which dual endothermic and exothermic peaks can be distinguished (Figure S19, Supporting Information). Such a bimodal behavior is ascribed to the crystallization process of *α*‐ and *β*‐form crystals of *N*‐22 induced by the heterogeneous and homogeneous nucleation, respectively. It should be noted that some DSC thermograms show multiple heat flow values at a single temperature point. This result is due to the occurrence of the crystallization of the *β*‐form crystal induced by the *α*‐form crystal as a metastable rotator phase during the crystallization process of *α*–form crystal. The crystallization of *β*‐form crystal can release a large amount of latent heat during a short period of time, leading to an increase in the temperature of *N*‐22 along with the multiple heat flow values in a single temperature point in the DSC thermograms. This phenomenon is usually found in the crystallization process of paraffins.^[^ ^31^ ^]^ The phase‐change temperatures, i.e., melting temperature (*T*_m_) and crystallization temperature (*T*_c_), were found to fluctuate in the different phase‐change composites (Figure S20 and Table S3, Supporting Information), indicating that the thermophysical properties of the impregnated *N*‐22 are influenced by the aerogel skeletons. Pure *N*‐22 was found to show slightly higher melting enthalpy (Δ*H*_m_) and crystallization enthalpy (Δ*H*_c_) in their phase‐change composites (Figure 5d; Table S3, Supporting Information). Such a decrease in the phase‐change enthalpies is due to the presence of the FPI aerogel skeletons without a latent heat‐storable function. Nevertheless, the OT‐46@*N*‐22 composite obtains a Δ*H*_m_ of 242.7 J g^−1^and a Δ*H*_c_ of 237.8 J g^−1^, which are higher than most of the paraffin‐based phase‐change composites reported in literature (Figure 5e), such as the PVDF/BN/PW composite,^[^ ^32^ ^]^ crown‐ether‐functionalized phase‐change microcapsules,^[^ ^33^ ^]^ polyaryloxyphosphazene/phosphorene foam/PW composite,^[^ ^34^ ^]^ graphene‐boron nitride phase‐change nonwoven,^[^ ^35^ ^]^ air‐dried graphene phase change composite,^[^ ^36^ ^]^ PA‐modified and zinc oxide‐filled wood/*N*‐22 composites,^[^ ^37^ ^]^ MF@*n*‐docosane composite,^[^ ^38^ ^]^ and Fe_3_O_4_‐functionalized κ‐carrageenan/melanin aerogel/*N*‐22 composite.^[^ ^39^ ^]^ Such high phase‐change enthalpies contribute to the high porosity and good hydrophobic performance of nondirectional FPI aerogels (Figures S21 and S22, Supporting Information). The high porosity facilitates the FPI aerogels to load more amount of *N*‐22, and the good hydrophobic performance is advantageous to the impregnation of *N*‐22 into the aerogels. With a superior latent heat‐storage capacity, the phase‐change composites are qualified as the upper layer for the dynamic temperature regulation through storing and releasing thermal energy by their loaded *N*‐22 PCM.

The thermal stability of the nondirectional FPI aerogel*/N*‐22 composites was evaluated by thermogravimetric analysis (TGA) to confirm the safety applications of the developed sandwich‐structured phase‐change composites. As seen in the TGA curve in Figure S23 (Supporting Information), both pure *N*‐22 and the FPI aerogel*/N*‐22 composites exhibit a typical one‐step thermal degradation behavior in the temperature range of 210–340 °C. The FPI aerogel*/N*‐22 composites show a characteristic temperature of 308 °C at around a maximum mass loss rate, which is in good agreement with that of pure *N*‐22 (304 °C). This result indicates the FPI aerogel has little influence on the thermal degradation of *N*‐22, enabling the FPI aerogel*/N*‐22 composites to meet a requirement for the application under high‐temperature operation conditions.

The dynamic temperature regulation performance of the nondirectional FPI aerogel/*N*‐22 composites was further investigated by an infrared thermographic technique using the ODA@*N*‐22, OT‐46@*N*‐22, and TFB@*N*‐22 composites as representative samples. Figure 5f,g shows the resultant infrared thermal images and corresponding temperature‐time evolution curves recorded on a hot plate at 180 °C. The three composite samples were found to show a slow rise in the surface temperature within 7 min, and their surface temperatures were all determined to be ≈45 °C after continuously heating for 7 min. It is importance to note the presence of temperature hysteresis due to the thermal energy absorption by the loaded *N*‐22 through a melting phase transition. With the completion of the melting phase transition, the surface temperature of the phase‐change composites tends to rise with an increase in heating time because of the sensible heat storage by the composites. These findings demonstrate that the sandwich‐structured composites can effectively prevent against a rise in their surface temperatures for a short period. Compared to the TFB@*N*‐22 composite, the ODA@*N*‐22 and OT‐46@*N*‐22 composites exhibit a longer period in their temperature hysteresis due to their higher latent heat‐storage capacity. In the cooling process, the three composite samples exhibit a significant reduction in their surface temperature within 6 min, and then their temperatures tend to be stable (Figure 5h,i). This is attributed to the sensible and latent heat release by the composites and their loaded *N*‐22. It should be noted that both the OT‐46‐based nondirectional FPI aerogel and OT‐46@*N*‐22 composite as two representatives show high infrared emissivity (Figure S24, Supporting Information). Such high infrared emissivity favors the thermal radiation of the phase‐change composite, thus accelerating the heat loss. As a result, the sensible heat release leads to a quick rise in the temperature, whereas it takes a long period for the stored latent heat to be released due to the low thermal conductance of the phase‐change composites. As seen in Figure S25 (Supporting Information), the OT‐46@*N*‐22 composite as a representative sample presents a low thermal conductivity of 122.35 mW m^−1^ K^−1^, which is much lower than that of pure *N*‐22. Such a low thermal conductivity can delay the heat transfer process when the stored latent heat is released.

**Figure 6:**
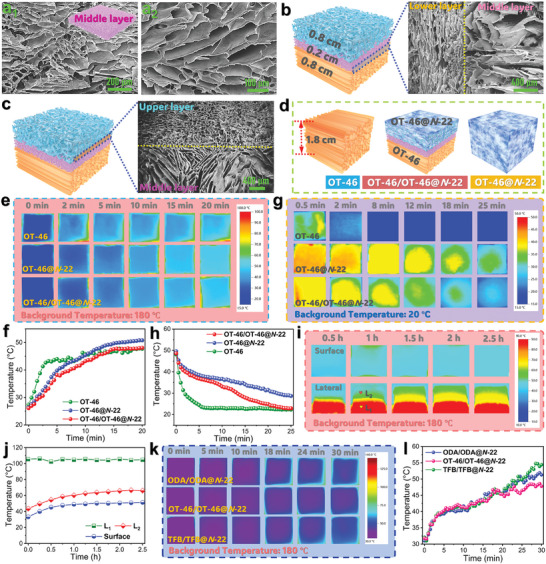
a) SEM images of ODA‐based PI aerogel obtained at a high solid content of PAA. b) SEM image of OT‐46‐based sandwich‐structured FPI aerogel at the boundary between the lower layer and middle layer. c) SEM image of OT‐46‐based sandwich‐structured FPI aerogel at the boundary between the upper layer and middle layer. d) Schematic structure for OT‐46‐based FPI aerogel, and OT‐46/OT‐46@*N*‐22 and OT‐46@*N*‐22 composites. e) Infrared thermal images and f) corresponded temperature‐time evolution curves of OT‐46‐based FPI aerogel, and OT‐46/OT‐46@*N*‐22 and OT‐46@*N*‐22 composites during heating at 180 °C. g) Infrared thermal images and h) corresponded temperature‐time evolution curves of OT‐46‐based FPI aerogel, and OT‐46/OT‐46@*N*‐22 and OT‐46@*N*‐22 composites during natural cooling process at 20 °C. i) Lateral and surface temperature distributions and j) corresponded temperature‐time evolution curves of OT‐46/OT‐46@*N*‐22 at different locations during heating at 180 °C. k) Infrared thermal images and l) corresponded temperature‐time evolution curves of ODA/ODA@*N*‐22, OT‐46/OT‐46@*N*‐22, and TFB/TFB@*N*‐22 during heating at 180 °C.

The nondirectional PI aerogel synthesized at a high solid content of PAA was investigated to confirm the microstructure of the middle layer in the sandwich‐structured aerogels. As observed from the SEM image in **Figure** **6**a, this PI aerogel was formed with a closed and opened porous structure due to a high solid content of PAA. It is expected that the high‐solid‐content PI aerogel exhibits a higher density than the low‐solid‐content one (Figure S26, Supporting Information). The molecular packing density of the frozen PAA block can be increased under a high solid content of PAA, suppressing the formation of an opened porous structure accordingly. As seen in Table S4 (Supporting Information), the porosity of this PI aerogel calculated from its density is higher than the result obtained from mercury intrusion porosimetry (Figure S27, Supporting Information). The ratio of opened pores was calculated to be 86.8%, confirming the existence of closed pores in the PI aerogel. It is worth noting that there is a distinct boundary between the two connected layers in the sandwich‐structured composite aerogel, demonstrating the different oriented porous structures of the connected layers (Figure 6b,c). Moreover, to balance the thermal insulation and dielectric properties of the sandwich‐structured composite, the thicknesses of upper, middle, and lower layers were optimized to be 0.8, 0.2, 0.8 cm according to the infrared thermographic analysis (Figure S28, Supporting Information).

After the sandwich‐structured composite was continuously heated at 100 °C for 12 h, the Δ*H*_m_ of the upper layer decreased by 16.8 J g^−1^ (Figure S29a, Supporting Information). In contrast, there is a significant reduction in the Δ*H*_m_ by 39.8 J g^−1^ after continuous heating for the double‐layered phase‐change composite with the nondirectional FPI aerogel/*N*‐22 composite upper layer and unidirectional FPI aerogel lower layer (Figure S29b, Supporting Information). Such a decrease in the Δ*H*_m_ is attributed to the large pore size of the upper layer of nondirectional aerogels (Figure S21, Supporting Information), which is disadvantageous to the confinement of the molten *N*‐22 during the heating process. To further confirm the positive influence of the middle layer on the antileakage of molten *N*‐22 from the upper layer to the lower one, the phase‐change enthalpies of the middle and lower layers after heating were characterized. The middle and lower layers show Δ*H*_m_’s of 13.1 and 1.2 J g^−1^, respectively (Figure S29c,d, Supporting Information). In contrast to the sandwich‐structured composite, the Δ*H*_m_ of the lower layer of the double‐layered composite was determined to be 36.2 J g^−1^ (Figure S29e, Supporting Information). This result is almost 30 times as much as that of the lower layer of the sandwich‐structured composite. These findings confirm the presence of a nondirectional PI aerogel middle layer can effectively prevent against the leakage of the molten *N*‐22 from the upper layer to the lower one in the sandwich‐structured composites. According to the results mentioned above, the antileakage behavior of the sandwich‐structured phase‐change composite can be described as follows: 1) The upper layer of the sandwich‐structured composite can act as the first barrier to prevent the leakage of *N*‐22 in the molten state effectively. 2) The different orientated porous structures of the connected layers act as the second barrier to increase the capillary forces between the two layers further to prevent against the exudation of the molten *N*‐22. 3) The middle layer of the sandwich‐structured composite contains closed pores, which can act as the third barrier to hinder the flow of the molten *N*‐22 from the upper layer to the lower one within the composite. 4) The capillary forces within the porous structure of the middle layer act as the last barrier to restrict the flow of the molten *N*‐22, impeding its movement toward to the lower layer of the composite.

To confirm the thermal cycling stability and leakage behavior of the upper layer of the sandwich‐structured phase‐change composites during the iterative heating–cooling process, a thermal cycling experiment was conducted using the OT‐46/OT‐46@*N*‐22 as a representative sample. The OT‐46/OT‐46@*N*‐22 composite was subjected to 100 thermal cycles by heating at 80 °C in an oven and cooling at 0 °C in a refrigerator. The phase‐change enthalpies of the OT‐46@*N*‐22 composite at every ten thermal cycles were measured by DSC. According to the DSC thermograms (Figure S30a, Supporting Information), the phase‐change enthalpies of the OT‐46@*N*‐22 composite decreased slightly during thermal cycles (Figure S30b, Supporting Information). Nevertheless, the OT‐46@*N*‐22 composite after thermal cycles was found to maintain phase‐change enthalpies as high as 222 J g^−1^, which only decreased by 3.1% compared to that before thermal cycles. This result suggests the good phase‐change reversibility and work durability of the OT‐46/OT‐46@*N*‐22 composite. As seen in the SEM images (Figure S31, Supporting Information), *N*‐22 is well confined within the porous structure of the nondirectional OT‐46 aerogel after thermal cycles. Compared to the OT‐46/OT‐46@*N*‐22 composite after one thermal cycle, the composite after 100 thermal cycles shows some frameworks in the nondirectional OT‐46 aerogel. This is ascribed to the leakage of a small amount of the molten *N*‐22, which is consistent with the results in Figure S30 (Supporting Information). These results further confirm that the middle and upper layers in the sandwich‐structured composite can prevent the leakage of most of the molten *N*‐22 during the thermal cycling process.

### 2.6 Thermal Insulation of Sandwich‐Structured Composites

Thermal insulation and electromagnetic wave‐transparent performance of the sandwich‐structured phase‐change composites were extensively investigated by using the OT‐46/OT‐46@*N*‐22 composite as a representative sample due to its better dielectric performance and higher latent heat capacity compared to other composite samples. Meanwhile, OT‐46‐based FPI aerogel and OT‐46@*N*‐22 composite were prepared as control samples under identical conditions, and these two controls were tailored to the same size as the OT‐46/OT‐46@*N*‐22 composite for thermal insulation assessment (Figure 6d). Figure 6e shows the infrared thermal images of the OT‐46‐based FPI aerogel, OT‐46@*N*‐22 composite, and OT‐46/OT‐46@*N*‐22 composite on a hot plate at 180 °C. These three samples were all found to exhibit an extremely slow growth rate in their surface temperature during heat, indicating their good thermal insulation performance. The surface temperatures of the OT‐46‐based FPI aerogel, OT‐46@*N*‐22 composite, and OT‐46/OT‐46@*N*‐22 composite only reached 48.1, 50.8, and 47.7 °C, respectively, after heating for 20 min (Figure 6f). Compared to the OT‐46‐based aerogel, the OT‐46@*N*‐22 and OT‐46/OT‐46@*N*‐22 composites show a slower increase in the surface temperature during heating within 5 min. It is understandable that the *N*‐22 loaded in these two phase‐change composites releases huge amounts of latent heat through a melting phase transition during heating, which can effectively prevent their surface temperatures from rising. Moreover, the OT‐46/OT‐46@*N*‐22 composite exhibits a lower surface temperature than the OT‐46@*N*‐22 composite. This can be attributed to the existence of the lower layer as a thermal insulation barrier to prevent against the heat transfer from the inside of the sandwich‐structured composite to the surface. These findings suggest that a layer‐by‐layer integration of unidirectional FPI aerogel and phase‐change composite enables the resultant sandwich‐structured composites not only to obtain a dynamic temperature regulation ability to deal with a short‐term heat impact but also to generate a long‐term thermal insulation effect on hot objects.

Figure 6g,h shows the infrared thermographic images and corresponded temperature revolution curves of the three samples in the cooling process with a background temperature of 20 °C. The sensible heat release by the OT‐46‐based FPI aerogel results in a fast decrease in the surface temperature. As for the OT‐46@*N*‐22 and OT‐46/OT‐46@*N*‐22 composites, there is distinct temperature hysteresis observed within the temperature range of 39.5−32.6 °C, which is caused by the latent heat release of the loaded *N*‐22. It should be noted that the OT‐46/OT‐46@*N*‐22 composite exhibits a shorter temperature hysteresis than the OT‐46@*N*‐22 one because of its smaller loading of *N*‐22, leading to less absorbed thermal energy. These results demonstrate that the sandwich‐structured composite will take a shorter time to recover its initial state ready for repetitive thermal insulation compared to conventional aerogel‐based phase‐change composites. To confirm the gradient thermal insulation capability of the sandwich‐structured composites, the lateral temperature and surface temperature of the OT‐46/OT‐46@*N*‐22 composite were recorded by using an infrared thermographic camera (Figure 6i). The marked location L_1_ exhibits a higher temperature than the location L_2_. After heating for 2.5 h, the surface temperatures of the locations L_1_ and L_2_ on the OT‐46/OT‐46@*N*‐22 composite are still maintained to be 104.8 and 66.2 °C, respectively (Figure 6j). This result demonstrates the gradient thermal insulation performance of the sandwich‐structured composites. With a unique nondirectional porous structure, the lower layer of the sandwich‐structured composites provides the first barrier against heat transfer from the lower layer to the surface, resulting in a sharp decrease in their surface temperature. Meanwhile, the *N*‐22 loaded within the upper layer of the composites can absorb huge amounts of thermal energy and store it as latent heat through a melting phase transition during heating, thus offering the second barrier to prevent the surface temperature from rising. In this case, the sandwich‐structured composites present a surface temperature of only 51.4 °C, which is much lower than the temperatures of the locations of L_1_ and L_2_. In addition, the surface temperature of the sandwich‐structured composite can remain stable all through the heating process within 2.5 h, suggesting that the developed sandwich‐structured composites have a long‐term thermal insulation ability at elevated temperatures. Figure 6k,l shows the infrared thermal images and corresponded temperature‐time revolution curves of the ODA/ODA@*N*‐22, OT‐46/OT‐46@*N*‐22, and TFB/TFB@*N*‐22 composites on a hot plate at 180 °C. These three sandwich‐structured composites exhibit a similar trend in their temperature evolution with time during the heating process, in which the OT‐46/OT‐46@*N*‐22 composite was found always to keep the lowest surface temperature among the three composites. After heating at 180 °C for 2.5 h, the ODA/ODA@*N*‐22, OT‐46/OT‐46@*N*‐22, and TFB/TFB@*N*‐22 composites still present surface temperatures of 51.3, 47.8, and 53.9 °C, respectively. This result can be attributed to the fact that the OT‐46/OT‐46@*N*‐22 composite possesses the highest porosity in its lower layer and the largest latent heat‐storage capacity for its upper layer among the three composites. This finding also confirms that high porosity and large latent heat‐storage capacity paly two critical roles in good thermal insulation.

### 2.7 Wave‐Transparent Performance of Sandwich‐Structured Composites

**Figure 7:**
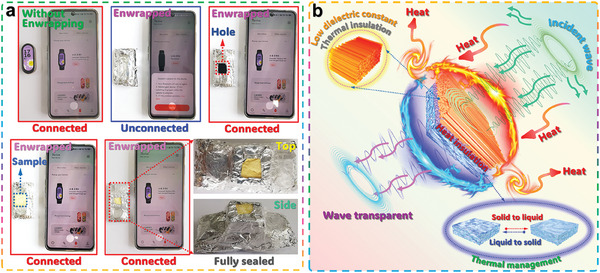
a) Digital photographs of electromagnetic wave transmission experiment for OT‐46/OT‐46@N‐22 composite (layer thickness: 0.8 mm for the upper layer, 0.2 mm for the middle layer, and 0.8 mm for the lower layer) through connecting a mobile phone and a wearable smart band by the Bluetooth. b) Schematic mechanism of sandwich‐structured composites for thermal protection and wave transmission.

The dielectric properties of the upper and middle layers of the OT‐46/OT‐46@*N*‐22 composites were characterized prior to the measurement of wave‐transparent performance of the sandwich‐structured phase‐change composites. Although the pores of the nondirectional PI aerogel have been filled with *N*‐22, the upper layer of the sandwich‐structured composite shows an average dielectric constant of 2.45 (Figure S32a, Supporting Information) in the frequency range of 8.2‒12.4 GHz, which is still lower than those of the conventional PI films reported in the literature.^[^ ^40^ ^]^ According to the Debye equation, the C─H and C─C bonds of *N*‐22 can reduce its polarizability and thus results in a low dielectric property of the sandwich‐structured composite. Moreover, the middle layer also shows a low dielectric constant in the frequency range of 8.9–12.4 GHz (Figure S32b, Supporting Information). These low dielectric properties enable the sandwich‐structured composites to obtain excellent wave‐transparent performance. The electromagnetic wave‐transparent performance of the developed sandwich‐structured composites was assessed by employing a mobile phone and a wearable smart band as tools in practical use. As shown in **Figure** **7**a, the wearable smart band can communicate with the mobile phone through the Bluetooth connection. When the wearable smart band is enwrapped with a tinfoil, it cannot communicate with the mobile phone due to the electromagnetic wave blocking by the tinfoil. The wearable smart band can connect with the mobile phone successfully when opening a hole on the tinfoil. After covering the hole with the developed sandwich‐structured composite, the connection between the wearable smart band and mobile phone can be well maintained by Bluetooth. This result indicates the good wave‐transparent performance of the sandwich‐structured composite at 2.4 GHz. In summary, a combination of the low dielectric lower, middle, and upper layers enables the sandwich‐structured composites to obtain good electromagnetic wave transmission. On the other hand, the lower unidirectional FPI aerogel layer with high porosity contributes to excellent heat insulation performance, whereas the *N*‐22 loaded within the middle layer contributes to a good dynamic temperature regulation capability through its phase transitions. This ensures the sandwich‐structured composites achieve good gradient thermal protection effectiveness (Figure 7b). As a result of the rational integration of these three functional layers with their respective structures and performance, the developed sandwich‐structured composites developed in this study exhibit immense potential for the simultaneous application of thermal insulation and electromagnetic wave transmission.

## 3 Conclusion

In conclusion, a novel type of bifunctional composite with a featured sandwich structure was successfully fabricated for the simultaneous applications of thermal protection and electromagnetic wave transmission. Such a sandwich‐structured composite is based on a unidirectional FPI aerogel lower layer, a high‐solid‐content nondirectional PI aerogel middle layer, and a nondirectional FPI aerogel/*N*‐22 composite upper layer. In this composite system, the lower layer exhibits a unique unidirectional porous structure along with a high porosity of 93.5% and an ultralow dielectric constant of 1.04. Owing to the introduction of the *N*‐22 as a PCM with a high latent heat‐storage capacity of 242.7 J g^−1^, the upper layer presents a dynamical temperature regulation capability for heat buffering. The nondirectional PI aerogel middle layer not only reveals low dielectric performance but also exhibits a good thermal insulation capability, and it can also prevent against the exudation and leakage of the molten *N*‐22 from the upper layer to the lower layer during heat buffering. Through a rational integration of these three functional layers, the resultant sandwich‐structured phase‐change composite can generate a prominent gradient thermal insulation effect on any hot objects, and it only presents a low surface temperature of 47.8 °C when placed on a hot plate at 180 °C for 2.5 h. Moreover, the sandwich‐structured composite shows an excellent wave‐transparent capability to establish communication between two electronic devices that are electromagnetically shielded. With such simultaneous thermal insulation and wave‐transparent functions, the sandwich‐structured composite developed by this work exhibits great potential for specific applications in aircraft, spacecraft, radar systems, and satellite communication. This study provides new insights for the development of advanced functional materials for the simultaneous applications of thermal protection and electromagnetic wave transmission.

## 4 Experimental Section

### Materials

BPDA, ODA, TFB, DMAc, and *N*‐22 were commercially provided by Shanghai Macklin Biochemical Co., Ltd., China. Triethylamine (TEA), acetone, and ethanol were purchased from Beijing Chemical Factory Co., Ltd., China. All of chemicals and reagents are of analytical grade and used as received without further purification.

### Preparation of PAA Solutions

A series of PAA solutions with solid contents of 3.0 and 8.0 wt.% at different mass ratios of ODA to TFB were synthesized prior to the fabrication of the sandwich‐structured phase‐change composites. In a typical procedure, a three‐necked round‐bottom flask equipped with a mechanical stirrer was charged with ODA (1.81 g), TFB (4.33 g), and DMAc (100 mL), and the mixture was stirred at room temperature to achieve an ODA solution. Then, the flake was moved to an ice‐water bath, followed by adding BPDA (6.68 g) into the flask under agitation for 3.0 h to obtain a clear solution. TEA (13.86 mL) was added dropwise into the flask under continuous agitation for 4.0 h to obtain a viscous PAA solution. Finally, excessive acetone was added into the viscous solution to obtain a PAA precipitate. The precipitated PAA were washed, filtrated, and dried at 70 °C for 36 h. In another round‐bottom flask, the precipitated PAA (1.5 g), TEA (1.0 mL), and deionized water (50.0 mL) were stirred continuously at room temperature for 5 h to obtain an OT‐46‐based PAA solution with a solid content of 3 wt.%.

### Fabrication of Sandwich‐Structured Phase‐Change Composites

A series of the sandwich‐structured aerogels were fabricated through directional freezing, multiple pouring freezing, freeze‐drying, and thermal imidization, followed by impregnating the molten *N*‐22 into the aerogels under vacuum conditions to form the sandwich‐structured composites according to the fabrication strategy depicted in Figure 1b,c. In a typical procedure, an OT‐46‐based PAA solution with a solid content of 3 wt.% was poured into a polytetrafluoroethylene mold with dimensions of 30 × 15 × 30 mm^3^ (length × width × height). The mold was placed on a copper platform and then immersed into liquid nitrogen to obtain a solidified PAA solution. The solidified PAA solution was placed horizontally in a silica gel mold with dimensions of 30 × 30 × 50 mm^3^ (length × width × height). The length and height of the solidified PAA solution correspond to the length and width of the silica gel to ensure the porous structure of the lower layer of the sandwich‐structured composite parallel to the horizontal plane. An ODA‐based PAA solution with a solid content of 8 wt.% was poured into the silica gel mold, and then the PAA solution was solidified in a refrigerator at −18 °C for 5 h. In succession, an OT‐46‐based PAA solution with a solid content of 3 wt. % was poured into the silica gel mold and solidified in the refrigerator at −18 °C for 5 h. The resultant solidified PAA sample was freeze‐dried to sublimate ice cylinders at −80 °C under a vacuum degree of 3–5 Pa for 72 h. The obtained sample was placed in a muffle furnace to perform thermal imidization at 150 °C for 1 h and at 300 °C for 2 h to obtain a sandwich‐structured aerogel. The upper layer of the resulting aerogel was immersed into the molten *N*‐22 at 80 °C in vacuum for 1 h, followed by natural cooling to room temperature. The OT‐46/OT‐46@*N*‐22 sandwich‐structured composite was obtained by removing the residual paraffin from the sample surface using filter paper.

### Characterizations and Measurements

Details for structural characterizations, performance measurements, and molecular and heat transfer simulations are presented in Sections S1−S3 (Supporting Information).

## Conflict of Interest

The authors declare no conflict of interest.

## Supporting information



Supporting Information

## Data Availability

Research data are not shared.
