# Supplementary material for: Sandwich‐Structured Fluorinated Polyimide Aerogel/Paraffin Phase‐Change Composites Simultaneously Enables Gradient Thermal Protection and Electromagnetic Wave Transmission
Source: Adv Sci (Weinh). 2024 Dec 6;12(5):2411758. doi: 10.1002/advs.202411758 (PMC11791978; doi:10.1002/advs.202411758)
Supplement: Supplementary file 1 — Supporting Information [file ADVS-12-2411758-s001.docx]

Supporting Information

**Sandwich-Structured Fluorinated Polyimide Aerogel/Paraffin Phase-Change Composites Simultaneously Enables Gradient Thermal Protection and Electromagnetic Wave Transmission**

*Tao Shi*^†^*, Jianwei Jing*^†^*, Zhiqiang Qian, Gaojie Wu, Guofeng Tian, Huan Liu*, and Xiaodong Wang**

**T. Shi, H. Liu, X. Wang**

State Key Laboratory of Organic–Inorganic Composites, Beijing University of Chemical Technology, Beijing 100029, China

E-mail: liu.huan@mail.buct.edu.cn (H. Liu); wangxd@mail.buct.edu.cn (X. Wang)

**J. Jing**

State Key Laboratory of Polymer Materials Engineering, College of Polymer Science and Engineering, Sichuan University, Chengdu, Sichuan 610065, China.

**Z. Qian**

Key Laboratory of Green and High-End Utilization of Salt Lake Resources, Qinghai Institute of Salt Lakes, Chinese Academy of Sciences, Qinghai Provincial Key Laboratory of Resources and Chemistry of Salt Lakes, Xining, Qinghai 810008, China

**G. Wu, G. Tian**

Key Laboratory of Carbon Fiber and Functional Polymers (Beijing University of Chemical Technology), Ministry of Education, Beijing 100029, China

^†^ The first two authors have contributed equally to this work.

**Section 1. Experimental**

***Characterizations and Measurements.*** The appearance of unidirectional fluorinated PI aerogels was recorded by a digital camera. The micro-morphologies of PI aerogels and phase-change composites were characterized by a scanning electron microscopy (SEM, Zeiss Gemini 300, Germany). The surface elemental composition and mapping images of unidirectional fluorinated PI aerogels were characterized by an energy-dispersive *X*-ray spectrometer (EDX, Oxford INCAX-Act, England). An *X*-ray photoelectron spectrometer (XPS, Thermo Fisher EscaLab 250Xi, USA) was performed to analyze the XPS spectra of unidirectional fluorinated PI aerogels using a focused monochromatized Al Ka radiation source. The chemical structure of unidirectional fluorinated PI aerogels was characterized by *Fourier*-transform infrared (FTIR) spectrometer (NicoletTM iS5, USA) in the wavelength range from 400 to 4000 cm^‒1^.

An electronic universal testing materials testing machine (CMT–4101, MTS Systems, USA) was used to characterize the mechanical properties of unidirectional fluorinated PI aerogels at a compression-loading rate of 5.0 mm min^–1^. The cyclic compression test was performed to evaluate the fatigue resistance of unidirectional fluorinated PI aerogels at a compression-loading rate of 5.0 mm min^–1^ until reaching 50 %. The mercury intrusion, pore size-distribution curves, porous parameters and densities of unidirectional fluorinated PI aerogels, ODA-based PI aerogel with a solid content of 8% and non-directional FPI aerogels was characterized by an automatic mercury porosimeter (Autopore V9620, Micromeritics Instrument, USA) with a pressure range of 0.5–62354.60 Psia. A thermal conductivity tester (HS-DR-5, Shanghai Hesheng Instruments, China) was performed to confirm the thermal conductivities of unidirectional fluorinated PI aerogels. The thermal stability of unidirectional fluorinated PI aerogels and *N*-22 phase-change composites was characterized by a thermogravimetric analyzer (TGA, Q50, TA Instruments, USA) at a heating rate of 10 °C min^–1^ under nitrogen atmosphere. The water contact angle of unidirectional fluorinated PI aerogels was characterized by a JC2000DM POWEREACH water contact angle measuring instrument. To determine the dielectric properties, the complex permittivity of PI aerogels and *N*-22 phase-change composite was measured by a waveguide method using a vector network analyzer (Anritsu, MS4644A, Japan). The unidirectional FPI aerogels was measured in the band of 2.5‒3.94 GHz, 3.94‒5.99 GHz, and 8.2‒12.4 GHz. The nondirectional ODA-based PI aerogel and OT-46@*N*-22 composite were measured in the band of 8.2‒12.4 GHz. Different sizes are required for the test samples at different frequency ranges when testing the dielectric properties. Specifically, the test samples measured in the band of 2.5‒3.94 GHz, 3.94‒5.99 GHz, and 8.2‒12.4 GHz are required to have the specific dimensions of 33.89 × 71.84 × 2.5, 22.0 × 47.25 × 2.5, and 22.9 × 10.2 × 2.5 mm^3^ (length × width × height), respectively. Therefore, a series of polytetrafluoroethylene molds with different dimensions of 35 × 75 × 2.5, 25 × 50 × 2.5, and 25 × 12 × 2.5 mm^3^ were prepared. The emissivity of OT-46-based non-directional FPI aerogel (upper layer) and OT-46@*N*-22 composite was characterized by an infrared spectrometer (Thermo Scientific Nicolet IS50, USA) with the wavelength range from 2.5 to 25 μm at a resolution of 8 cm^‒1^.

The phase-change behaviors of pure *N*-22 and phase-change composites were characterized by a differential scanning calorimeter (DSC, Q20, TA Instruments, USA) with a scanning rate of 10.0 °C min^–1^ under nitrogen atmosphere. The phase-change enthalpies were calculated by the TA Universal analysis software. The phase-change temperatures were directly achieved from the DSC thermograms. The thermal cycling stability of phase-change composites was also characterized by the DSC measurement. The thermal insulation and temperature management of unidirectional fluorinated aerogels, phase-change composites, and sandwich-structured composites were characterized by a thermal infrared imager (Testo 875-1i, Germany). The temperature distributions of the composites were analyzed by the Testo^TM^ ComSoft Basic software. Before the formal test, the infrared emissivity of the imager was set to be in accordance with the infrared emissivity of the samples. The temperature evolutions of unidirectional fluorinated aerogels were also characterized by a *k*-type thermocouple to confirm the reliability of the temperature results recorded by the thermal infrared imager.

**Section 2. Molecular simulation of fluorinated PI.**

Molecular dynamics simulation was performed to investigate the fraction free volume of fluorinated PI using the Materials Studio 8.0. The amorphous cell construction and molecular dynamics (MD) simulation were performed using COMPASS force field. A series of MD runs according to the previous work were carried out for dynamic equilibrium. In a typical simulation procedure, 1) 20-ps MD in the NPT ensemble at 0.5 GPa and 298 K was carried out to compress the model to experimental density; 2) Annealing the model by subsequent MD runs in the NVT ensemble at 598 K and 298 K, each about 20 ps; 3) Relaxing the model by a 20-ps NPT-MD at 0.0001 GPa and 298 K, checking the course of the density ﬂuctuations. The compression cycle should be repeated with a longer NPT-MD if the density-time curve was not stable; 4) Equilibrating the cell by a 300-ps NVT-MD run to further improve the equilibration. The fraction free volume (*φ*) of fluorinated PI can be calculated by the occupied volume (*V_o_*) and free volume (*V_f_*) based on the following equation:

(S1)

**Section 3. Heat transfer simulation of PI aerogel.**

The heat transfer of PI aerogel perpendicular and parallel to channels was simulated by the finite element software COMSOL Multiphysics. The solid heat transfer physics module was used to simulate the temperature evolutions as a function of heating time in the PI aerogel. A geometric model was constructed with the solid material consisted of a multilayer structure and an irregular concave-convex shape. Transient solution method was carried out for the simulation model with the same loading sub-steps of 11 steps and total time of 10 min. During the simulation analysis, the ambient temperature was set as 22 °C, the bottom and side surfaces of the aerogel were used as the heating surfaces, and the heating temperature was set as 180 °C. The convective heat flux on the aerogel surface, heat sources, and heat flux boundary conditions were considered. The solid heat transfer in the aerogel is governed by the heat conduction equations:

$\rho C_{p}\mathbf{u}\cdot\nabla T+\nabla\cdot\mathbf{q}=Q+Q_{ted}$ (S2)

$\mathbf{q}=-k\nabla T$ (S3)

where *ρ* represents density (kg m^–3^), *C_p_* is specific heat capacity at constant pressure (J kg^–1^ K^–1^), **u** is velocity vector (m s^–1^), **q** represents heat flux per unit area per unit time (W m^–2^), *Q* is the heat source term (W m^–3^), *Q_ted_* represents the heat source correction term (W m^–3^), and *k* represents the thermal conductivity (W m^–1^ K^–1^).


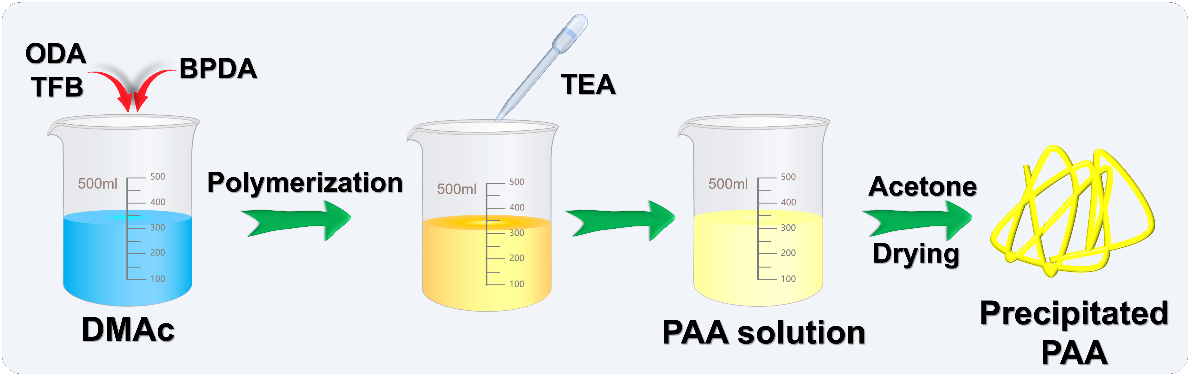


**Figure S1.** Scheme for the synthetic process of PAA.

**
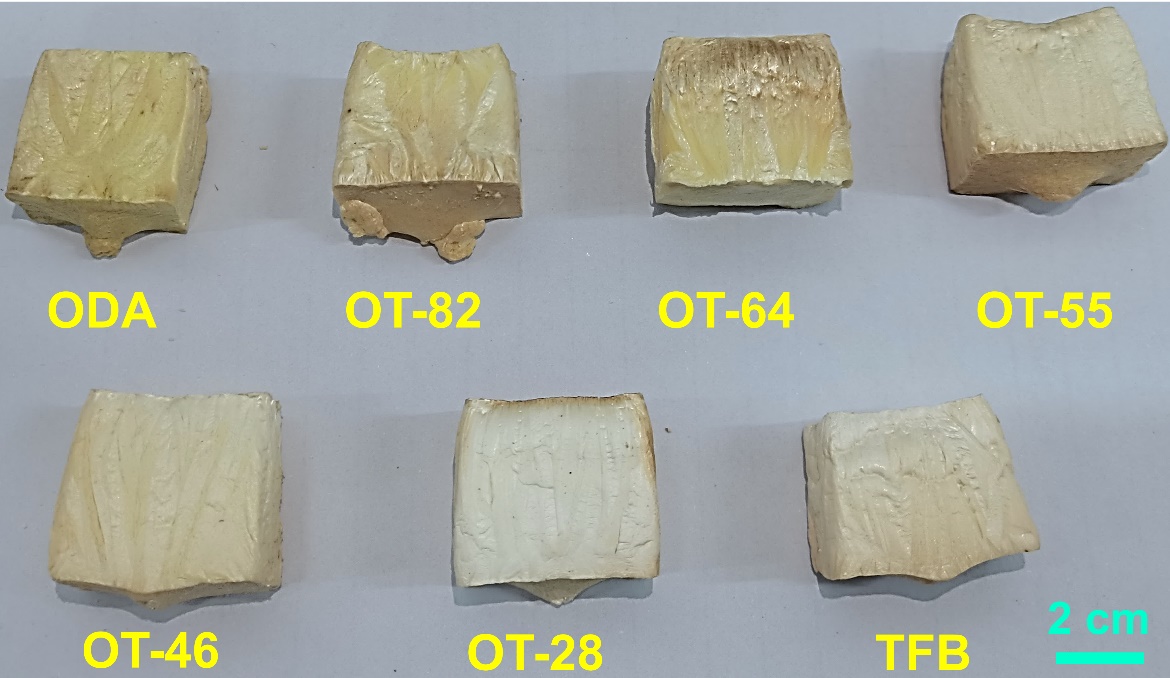
**

**Figure S2.** Digital photograph of unidirectional FPI aerogels with different contents of TFB.

**
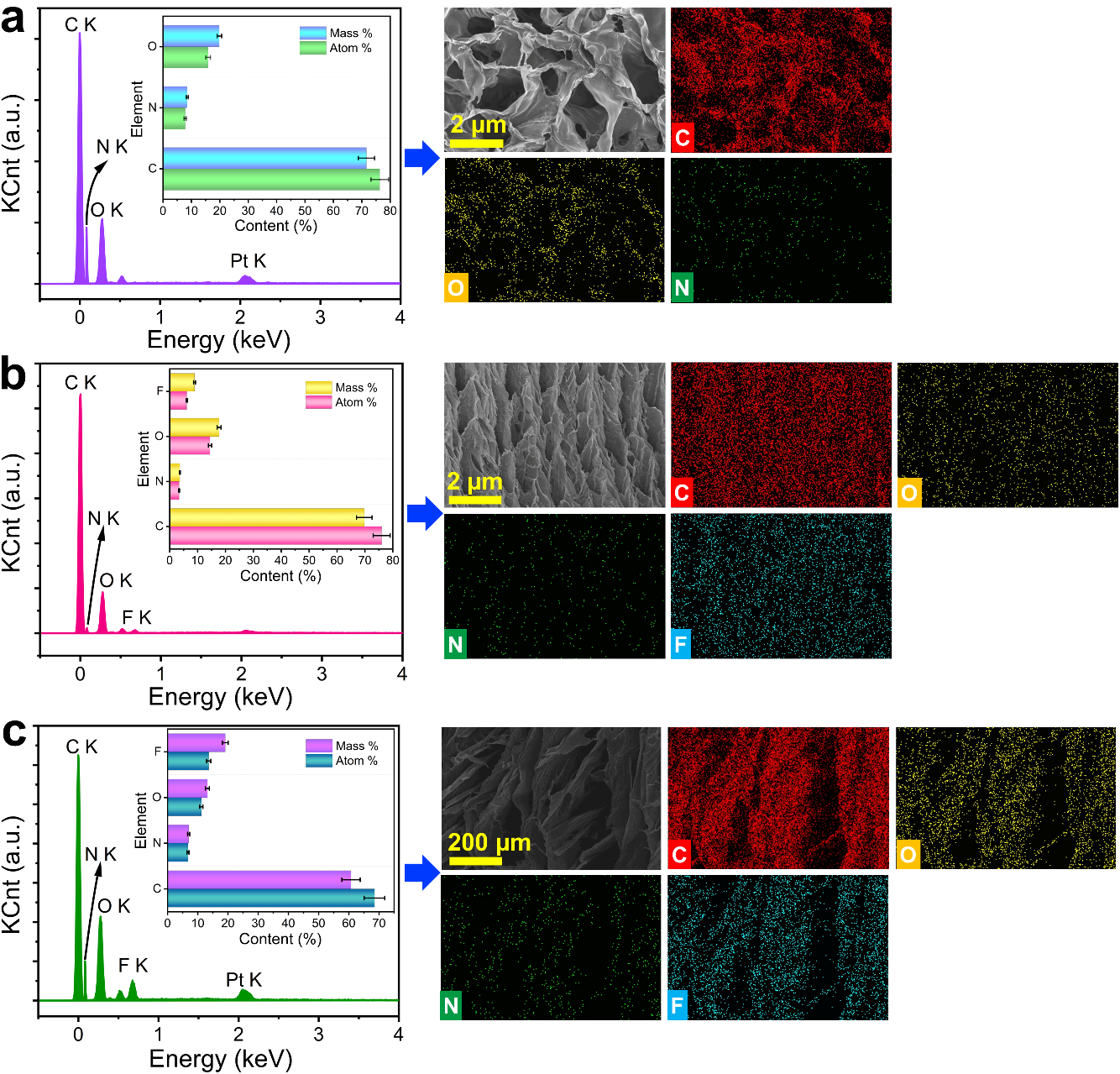
**

**Figure S3.** EDX spectra and corresponding elemental mapping images of (a) ODA-based, (b) OT-55-based, and (c) TFB-based FPI aerogels.


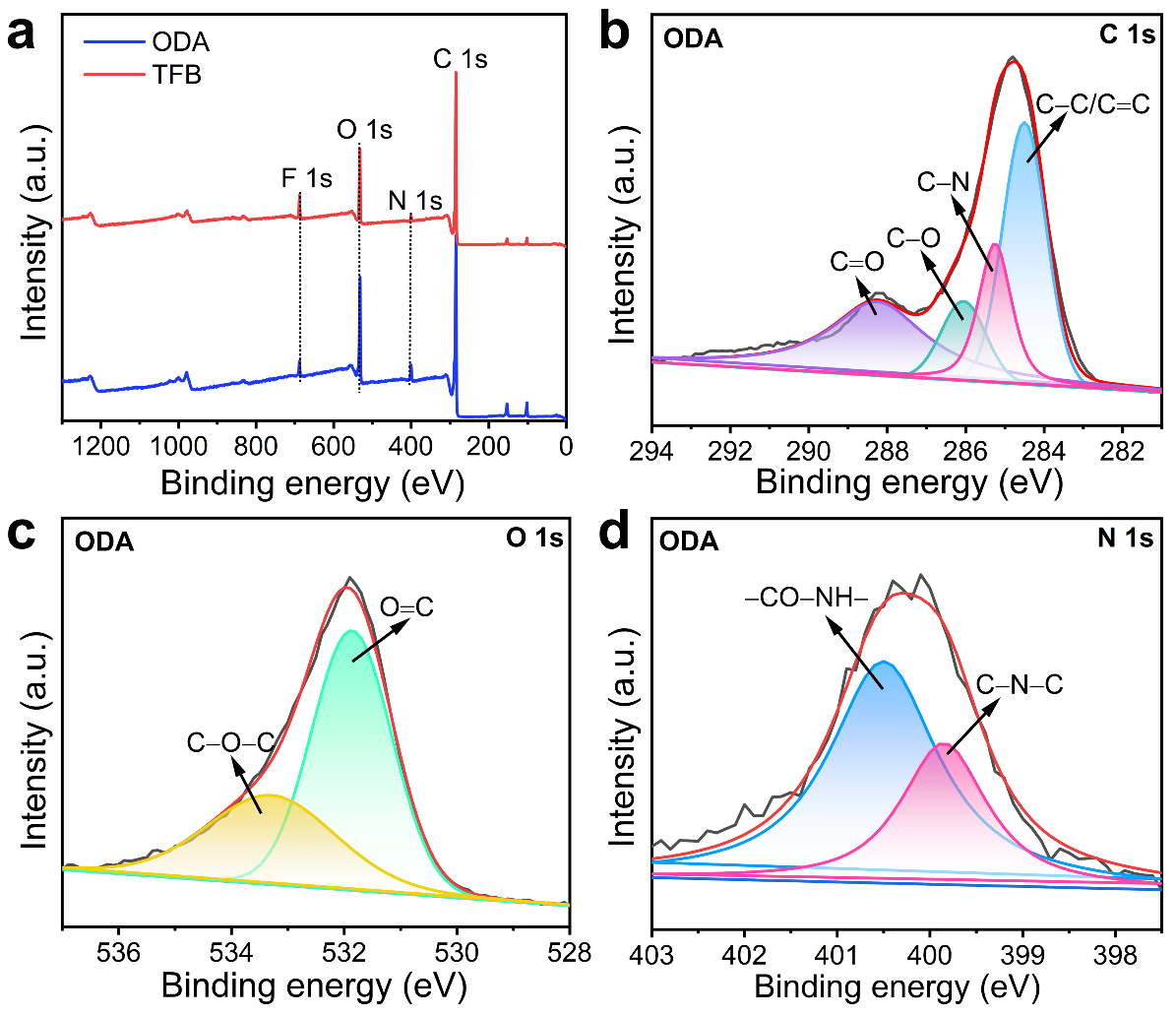


**Figure S4.** (a) Survey XPS spectra of ODA-based and TFB-based PI aerogels. (b–d) High-resolution XPS core-level spectra of ODA-based PI aerogel in binding energy ranges of (b) C 1s, (c) O 1s, and (d) N 1s.


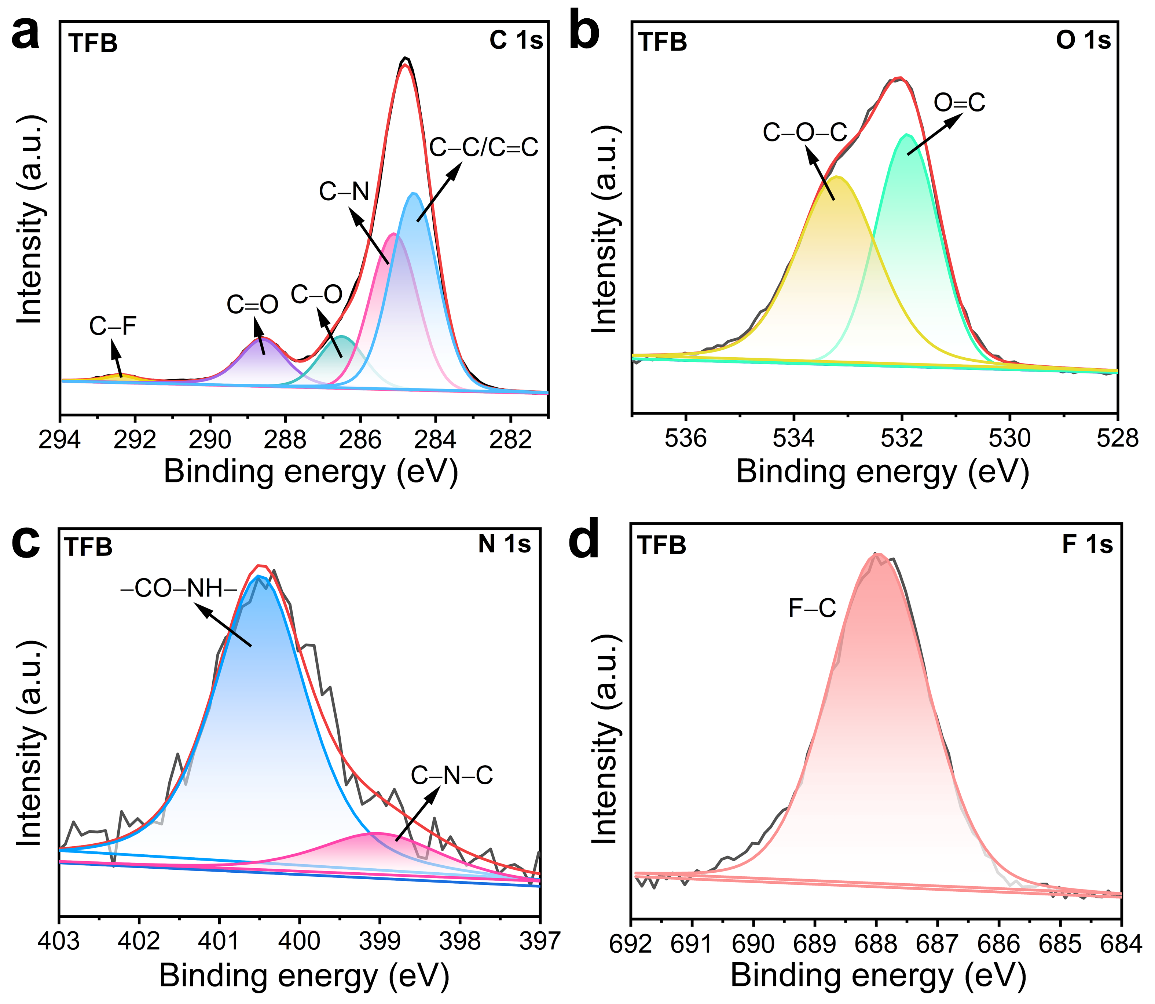


**Figure S5**. High-resolution XPS core-level spectra of TFB-based FPI aerogel in binding energy ranges of (a) C 1s, (b) O 1s, (c) N 1s, and (d) F 1s.

**
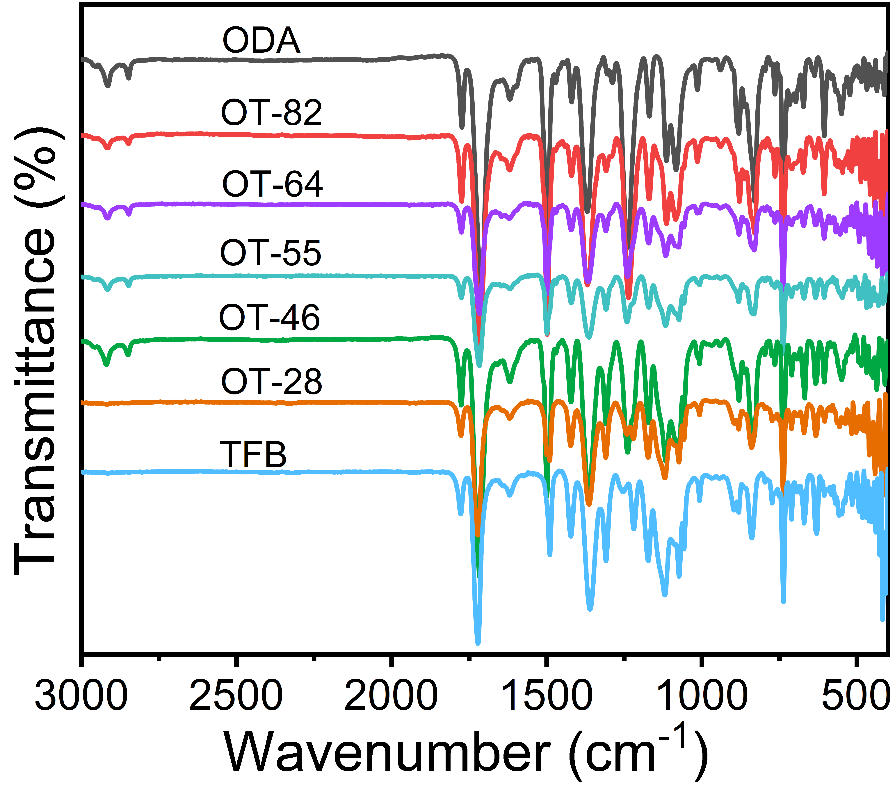
**

**Figure S6.** FTIR spectra of unidirectional FPI aerogels with different contents of TFB.

**
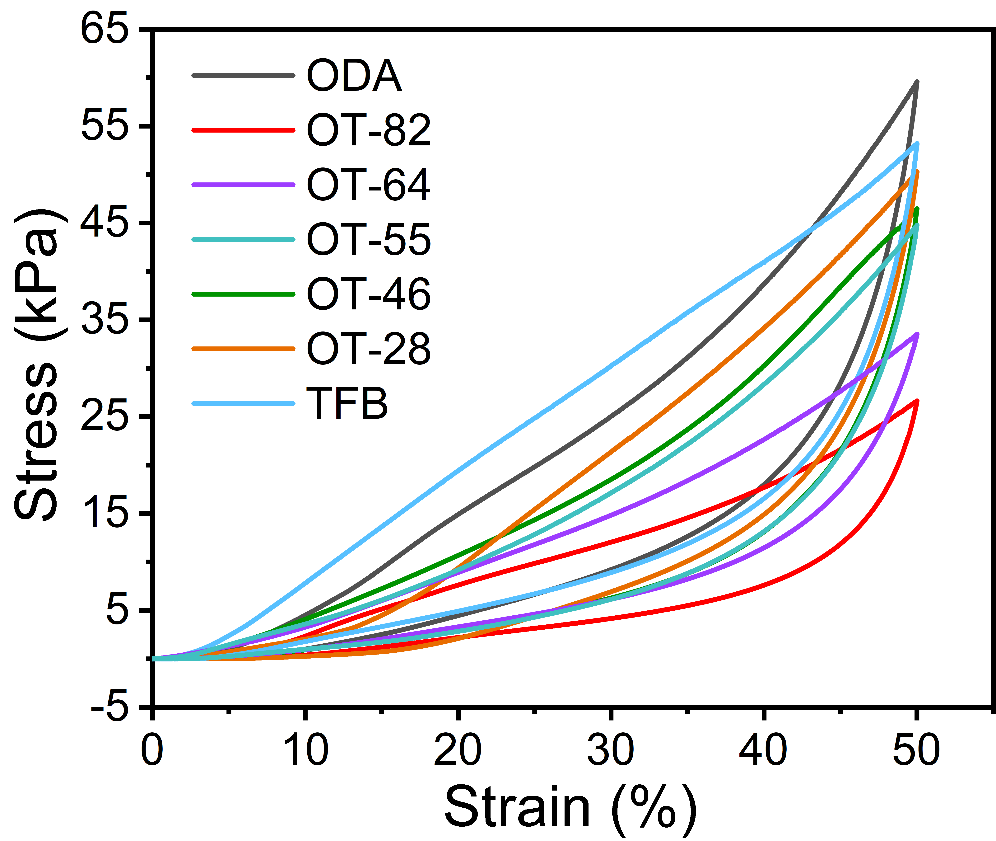
**

**Figure S7.** Stress–strain curves of unidirectional FPI aerogels with different contents of TFB.


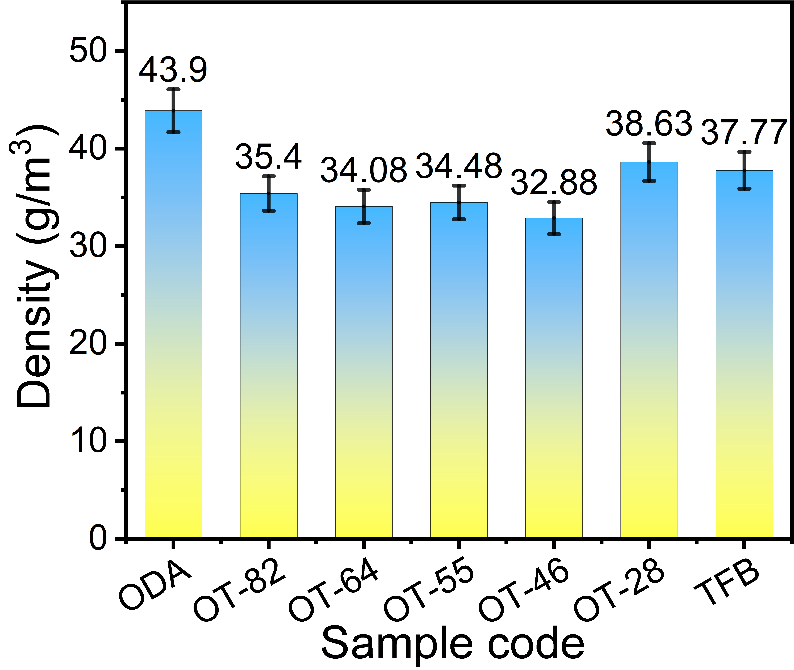

**Figure S8.** Densities of unidirectional FPI aerogels with different contents of TFB.


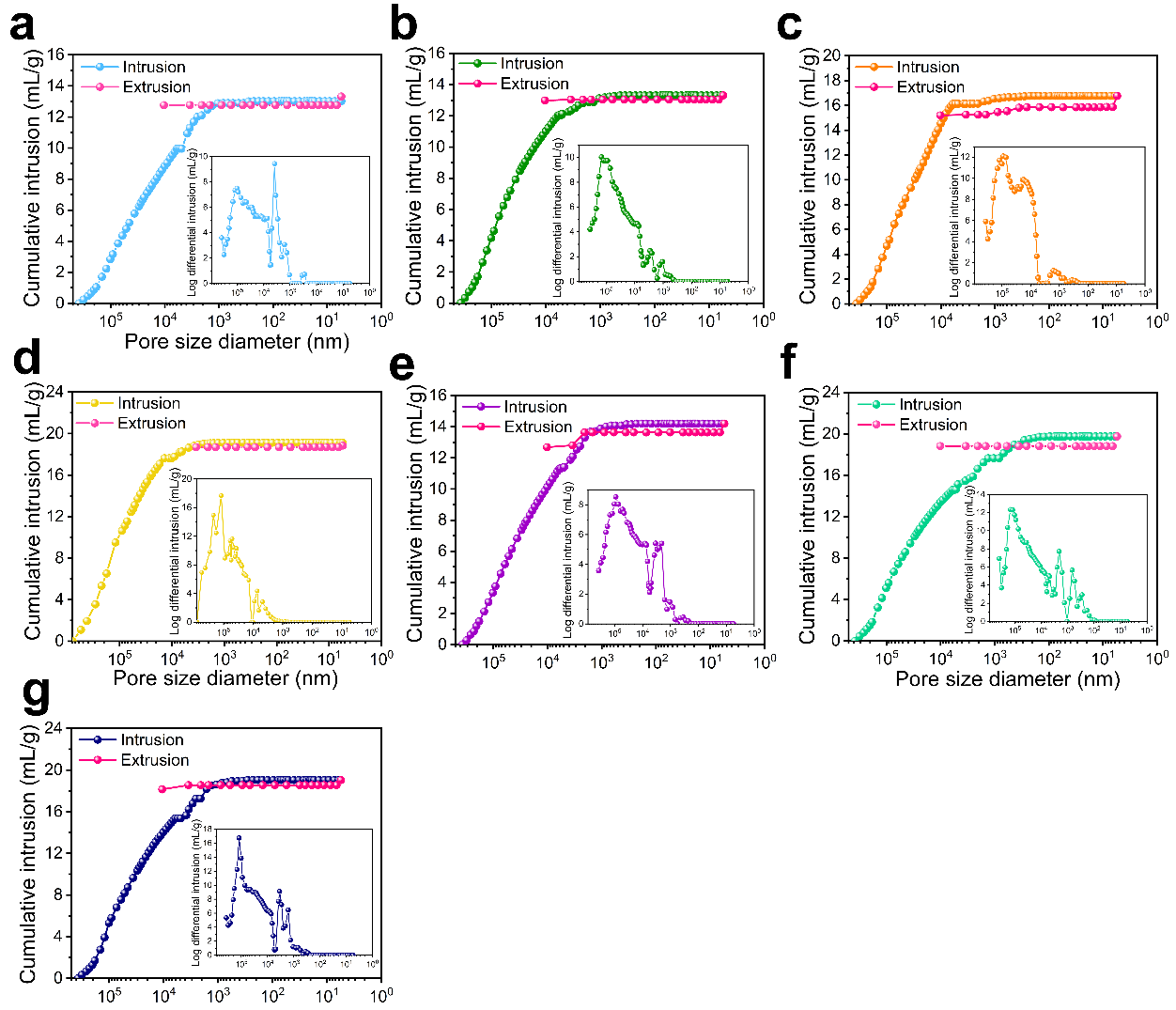


**Figure S9.** Mercury intrusion curves and pore size-distribution curves (inset) of a) ODA-based PI aerogel, b) OT-82-based, c) OT-64-based, d) OT-55-based, e) OT-46-based, f) OT-28-based, and g) TFB-based FPI aerogels.

**
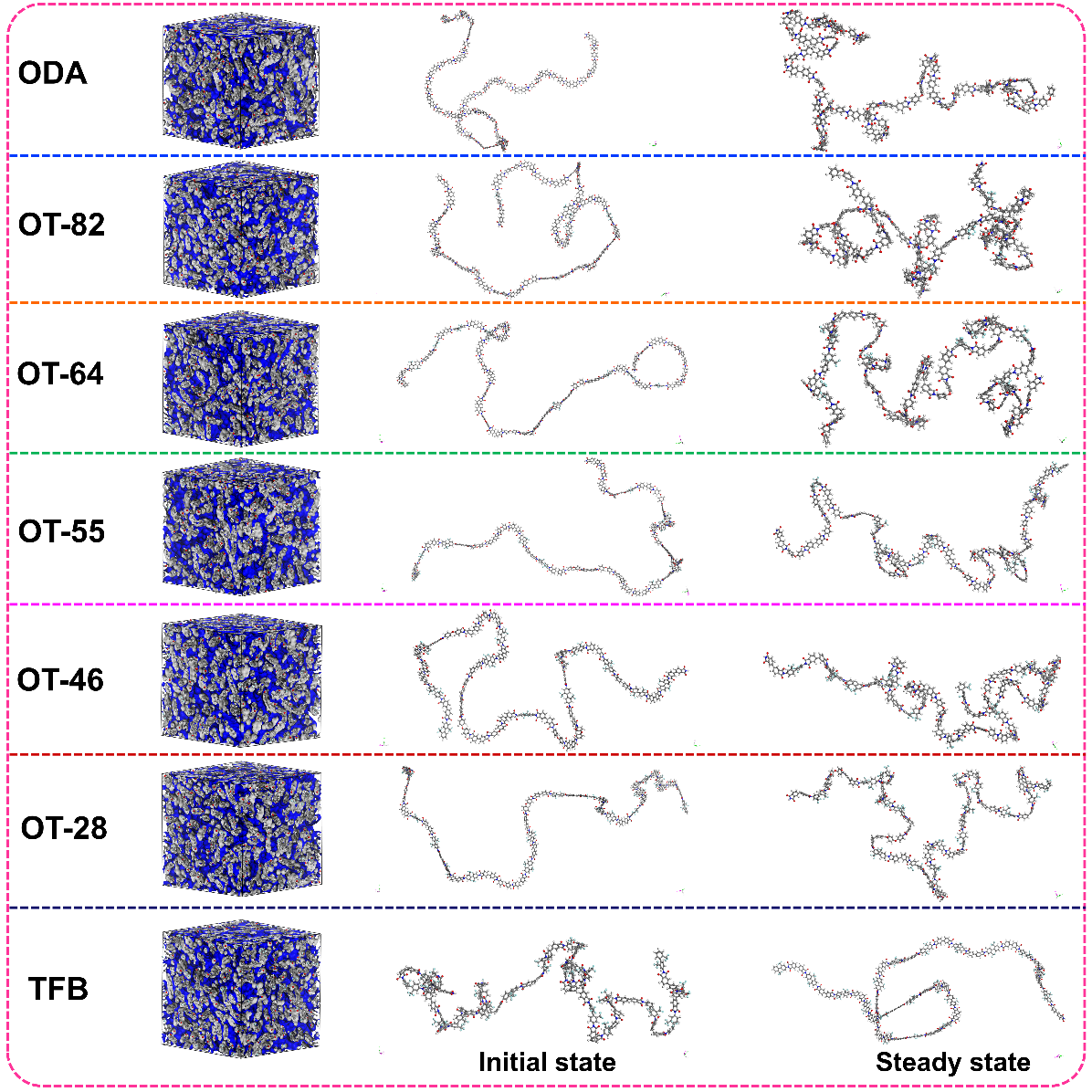
**

**Figure S10.** Simulation models and correspondung initial and steady states of PI molecules in the unidirectional FPI aerogels with different contents of TFB.

**
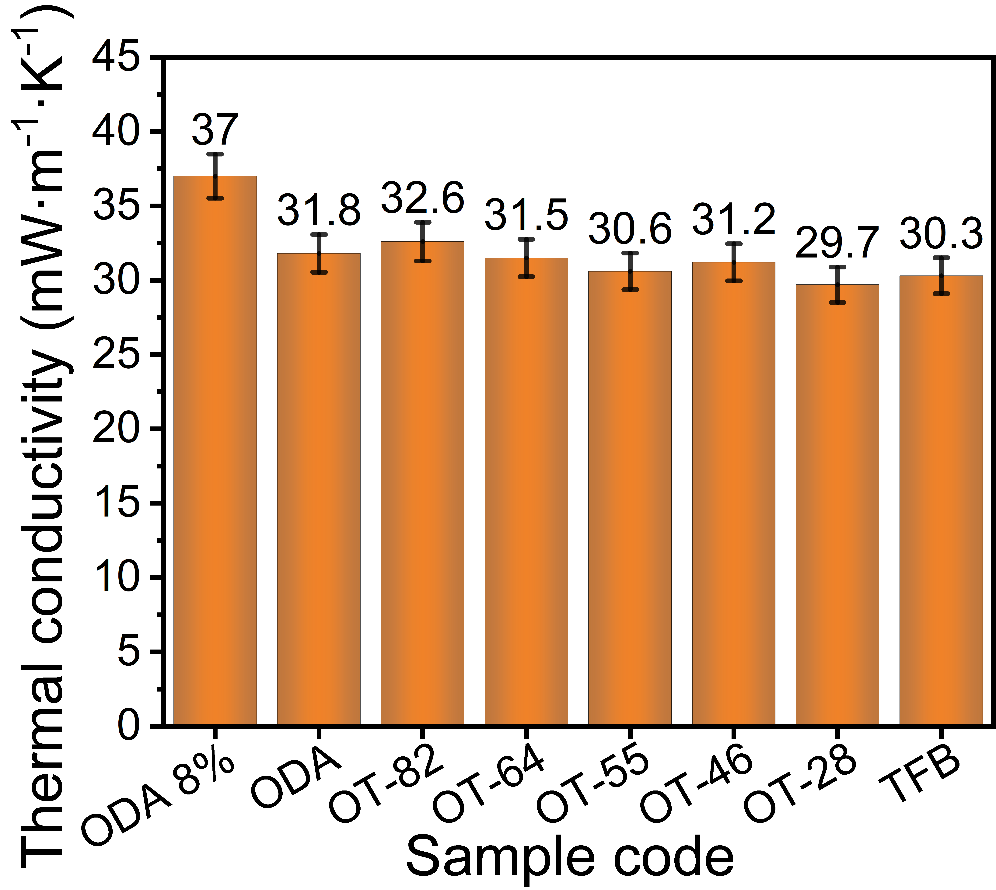
**

**Figure S11.** Thermal conductivities of various PI and FPI aerogel samples.

**
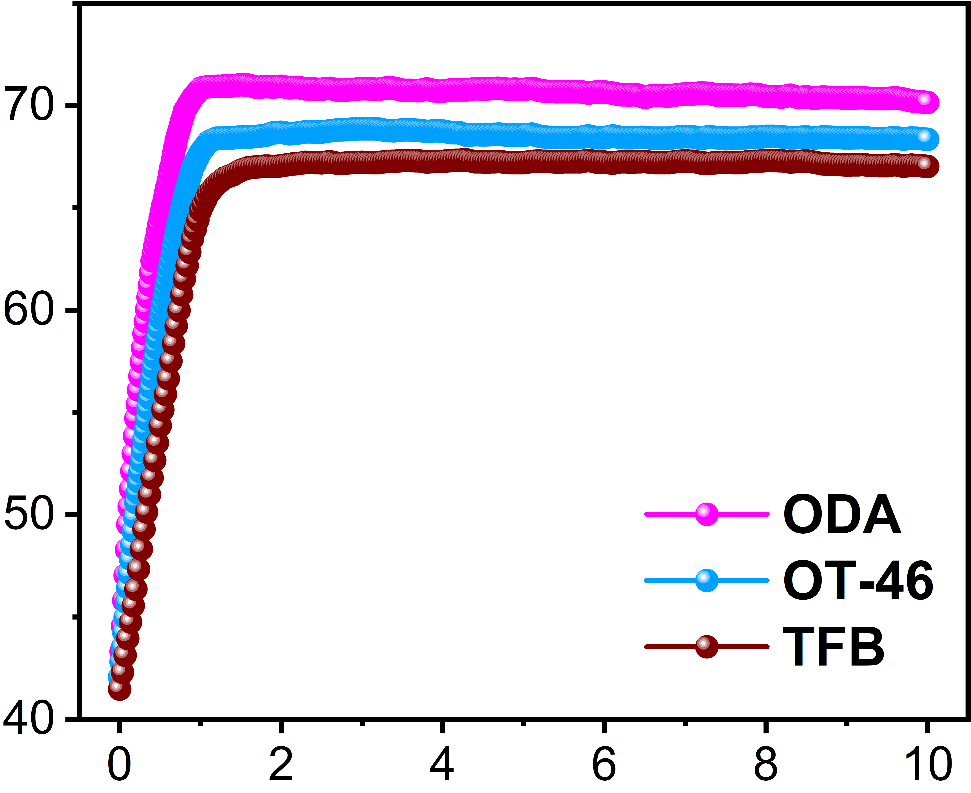
**

**Figure S12.** Temperature-time evolutions of ODA-based PI aerogel, and OT-46-based and TFB-based FPI aerogels during heating at 180 °C (recorded by thermocouples).

**
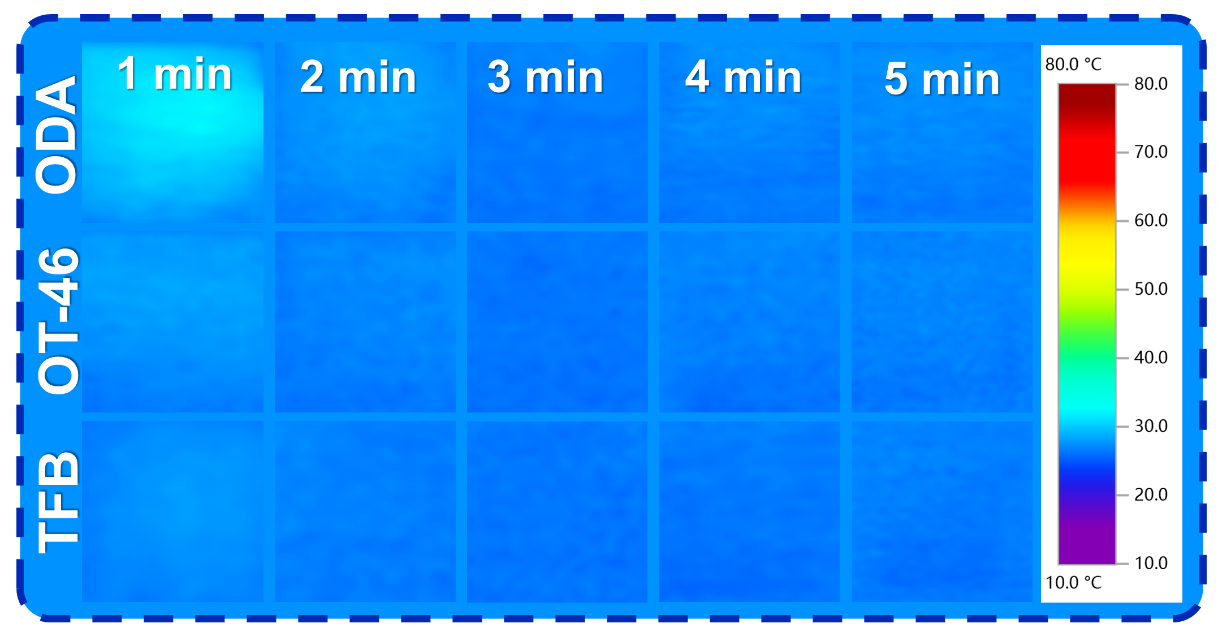
**

**Figure S13.** Representative infrared thermal images of ODA-based, OT-46-based, and TFB-based FPI aerogels in the cooling process.


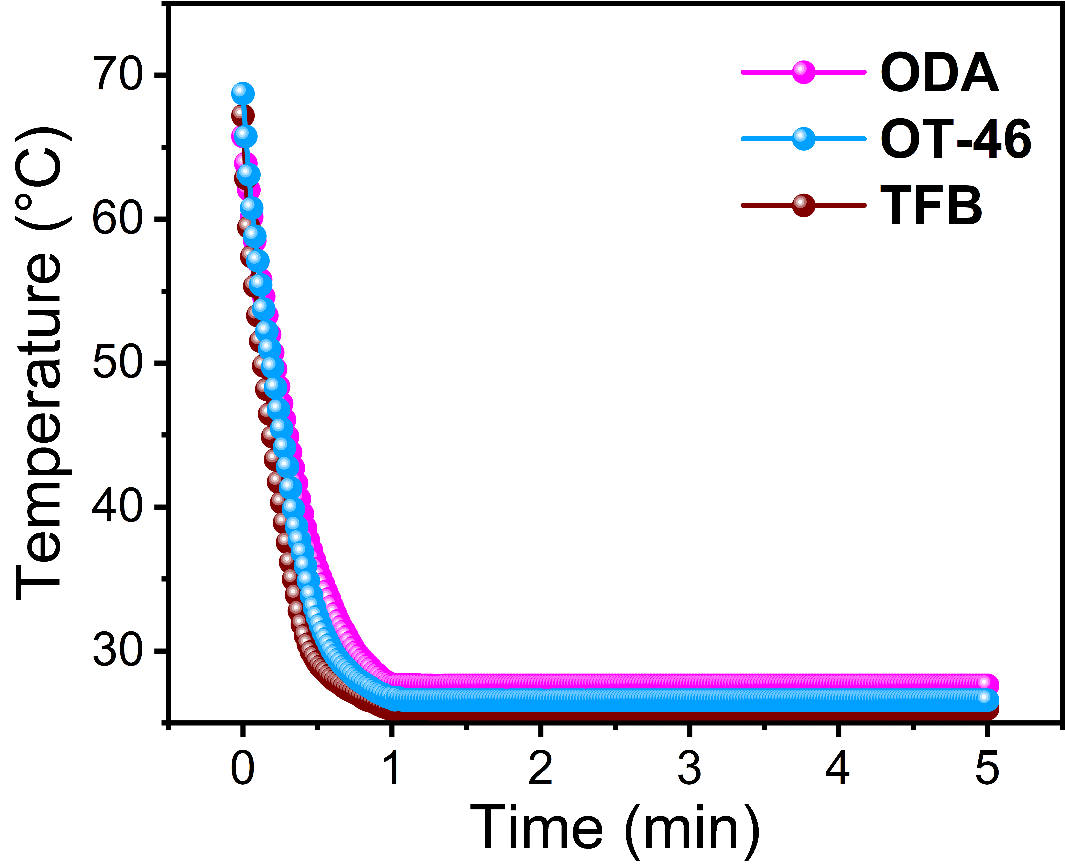


**Figure S14.** Temperature-time evolutions of ODA-based PI aerogel, and OT-46-based and TFB-based FPI aerogels in the cooling process (recorded by thermocouples).


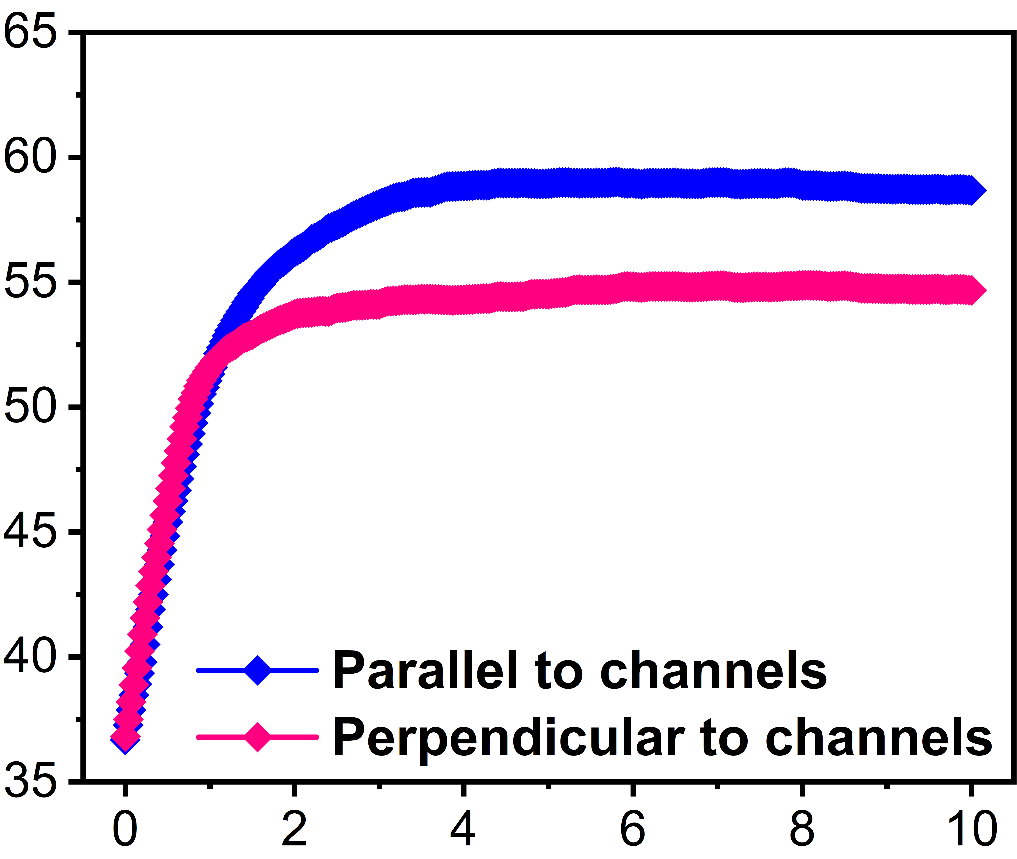


**Figure S15.** Temperature-time evolutions of the surface of OT-46-based FPI aerogel perpendicular and parallel to the channels during heating at 180 ºC (recorded by thermocouples).

**
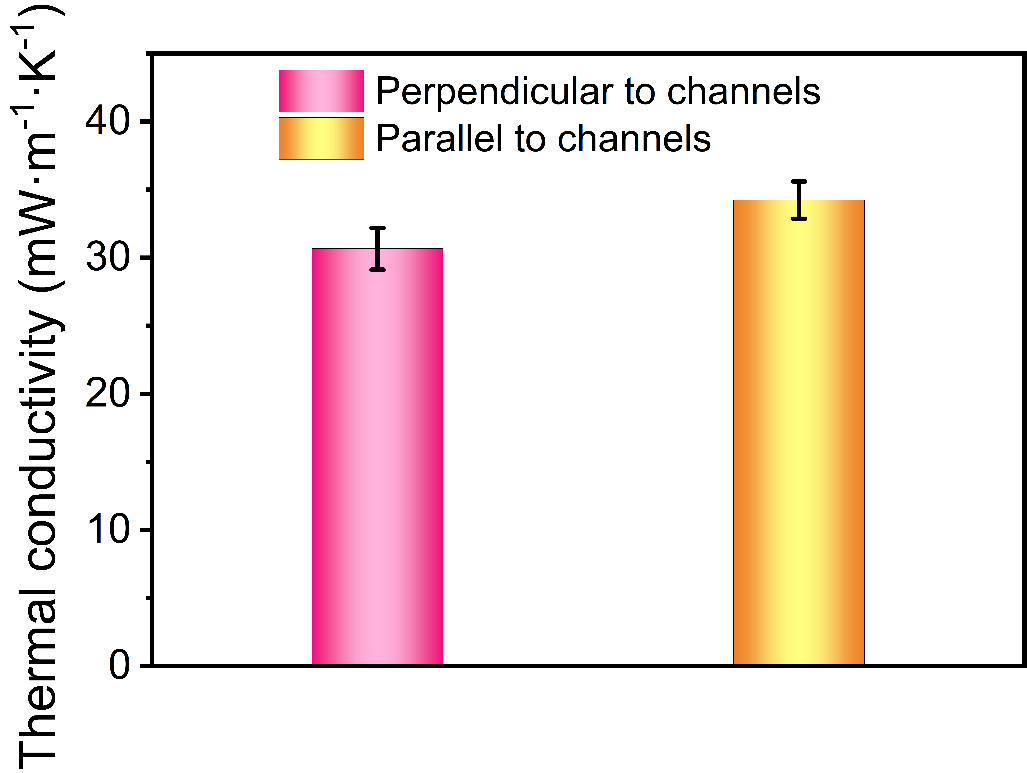
**

**Figure S16.** Thermal conductivities of OT-46-based FPI aerogel perpendicular and parallel to channels.

**
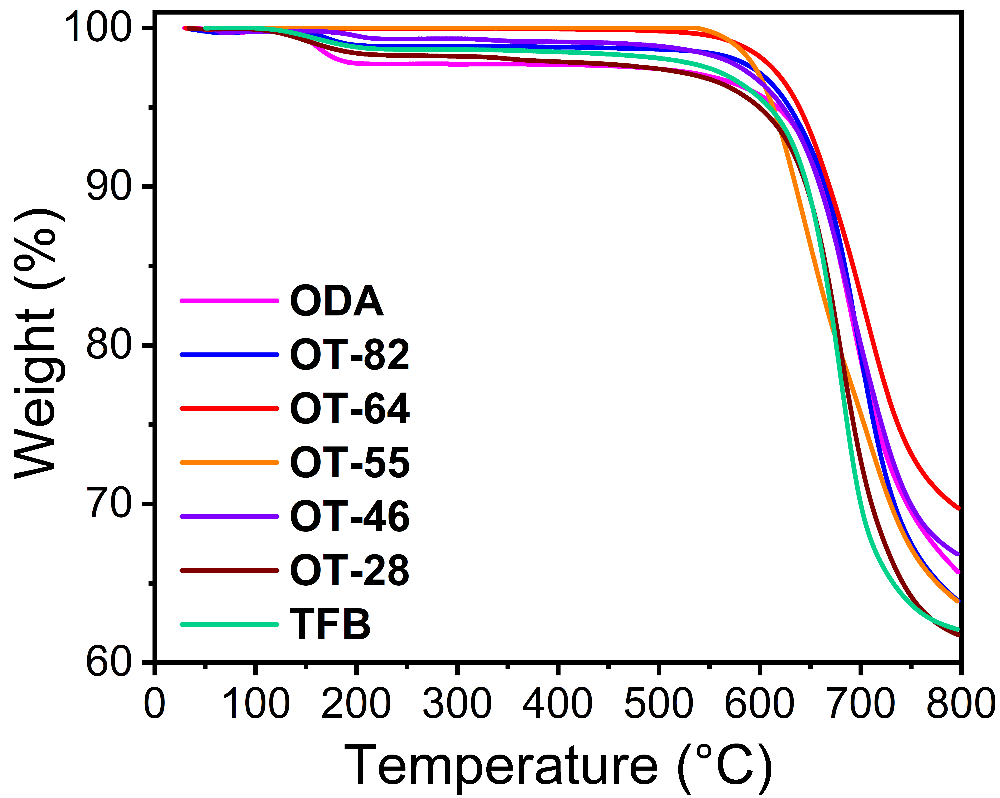
**

**Figure S17.** TGA thermograms of unidirectional FPI aerogels with different contents of TFB.

**
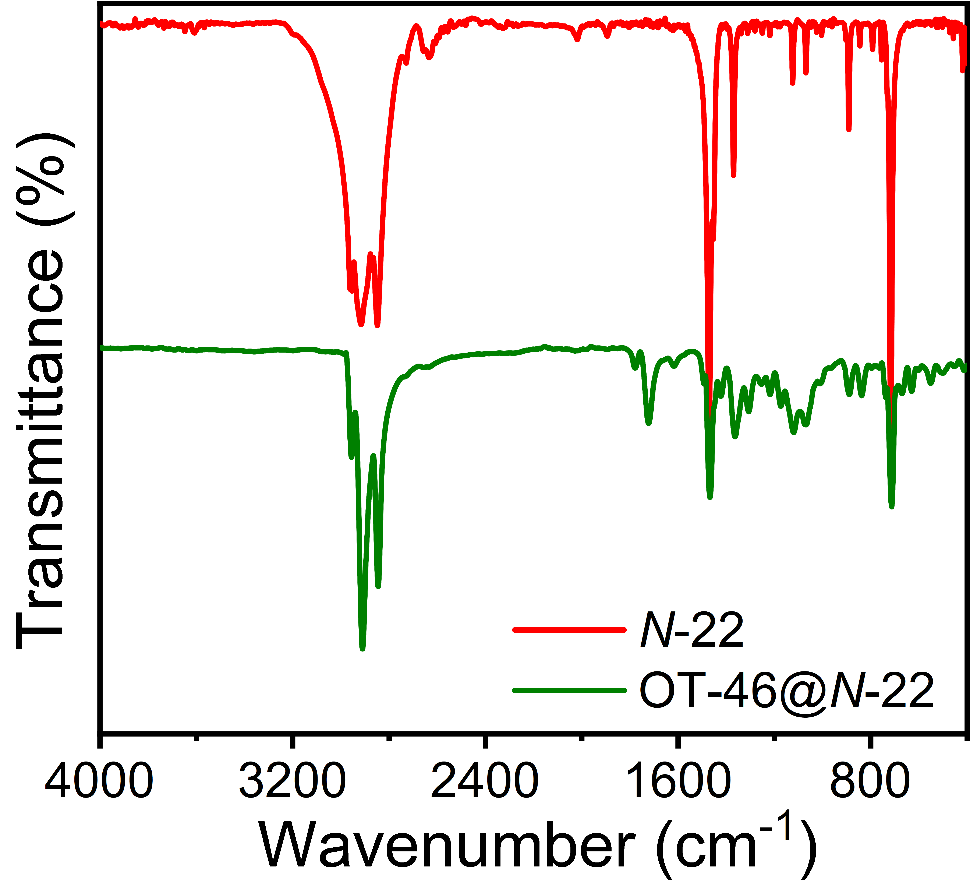
**

**Figure S18.** FTIR spectra of pure *N*-22 and OT-46@*N*-22 composite.

**
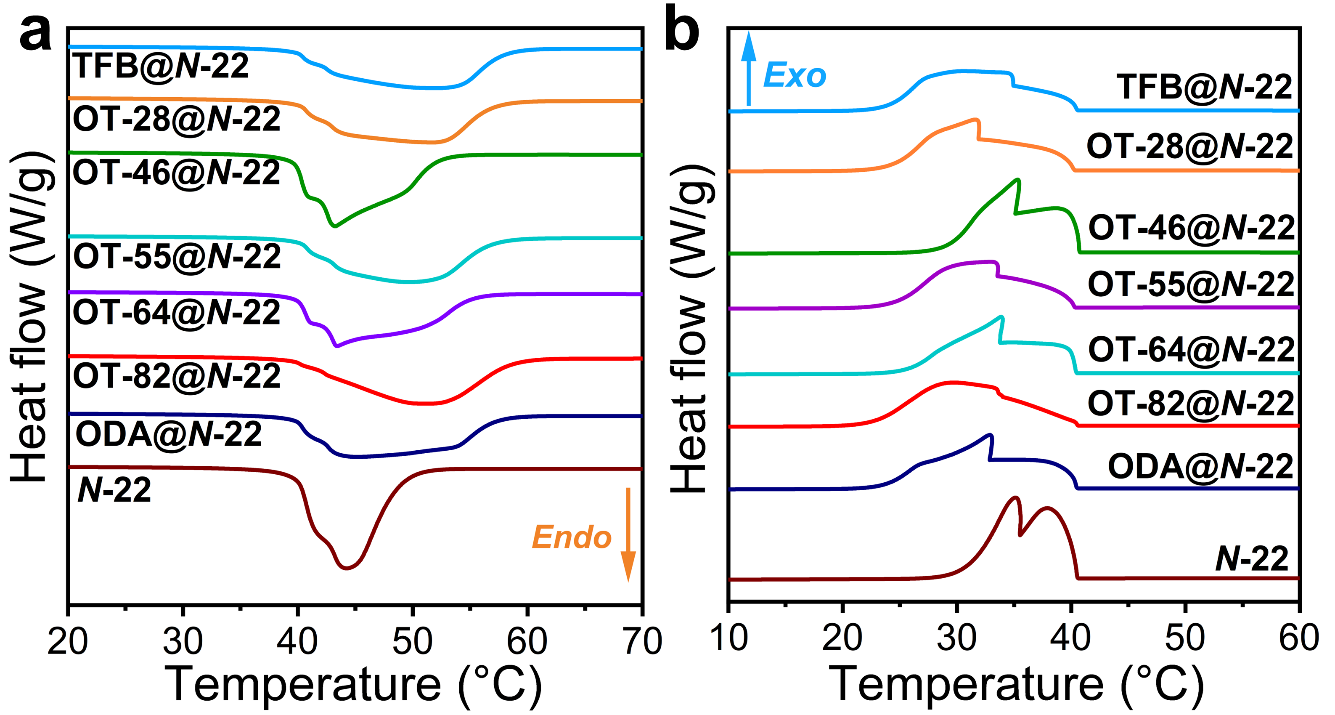
**

**Figure S19.** (a) DSC heating and (b) cooling thermograms of pure *N*-22 and PI aerogel@*N*-22 composites.

**
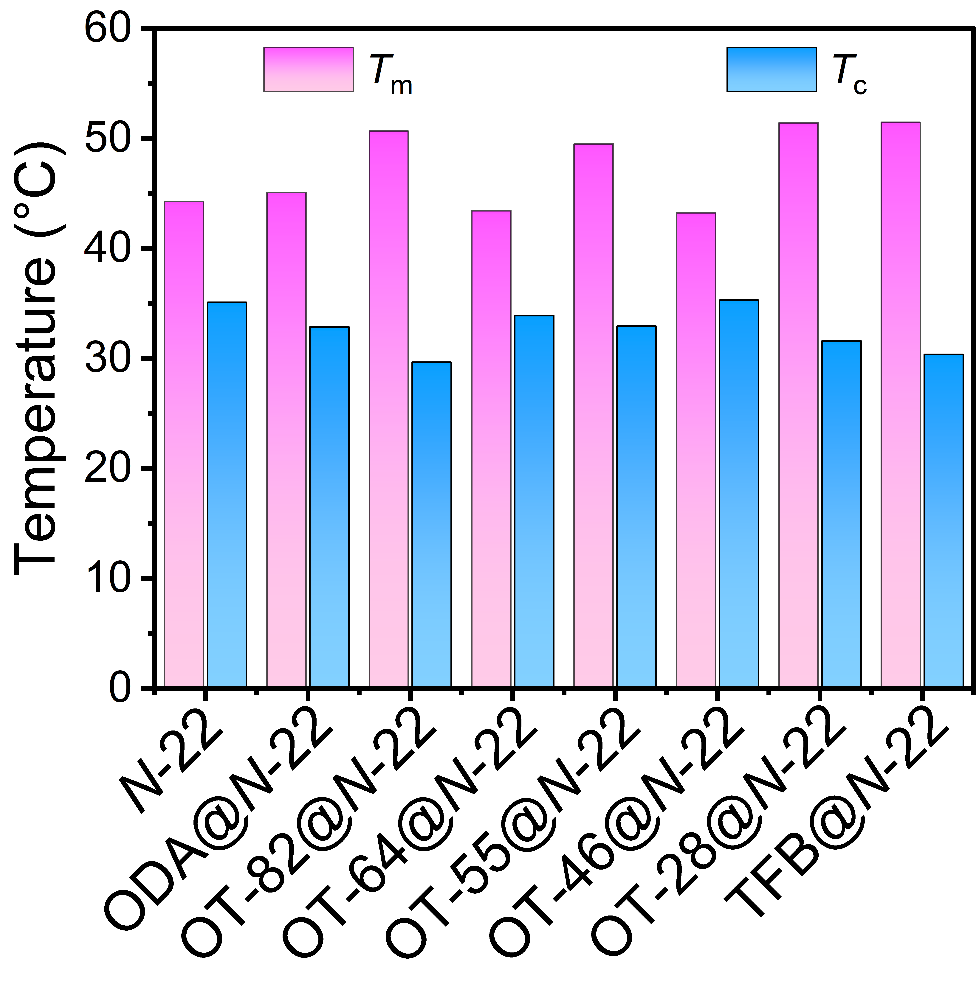
**

**Figure S20.** Phase-change temperatures of pure *N*-22 and PI aerogel@*N*-22 composites.


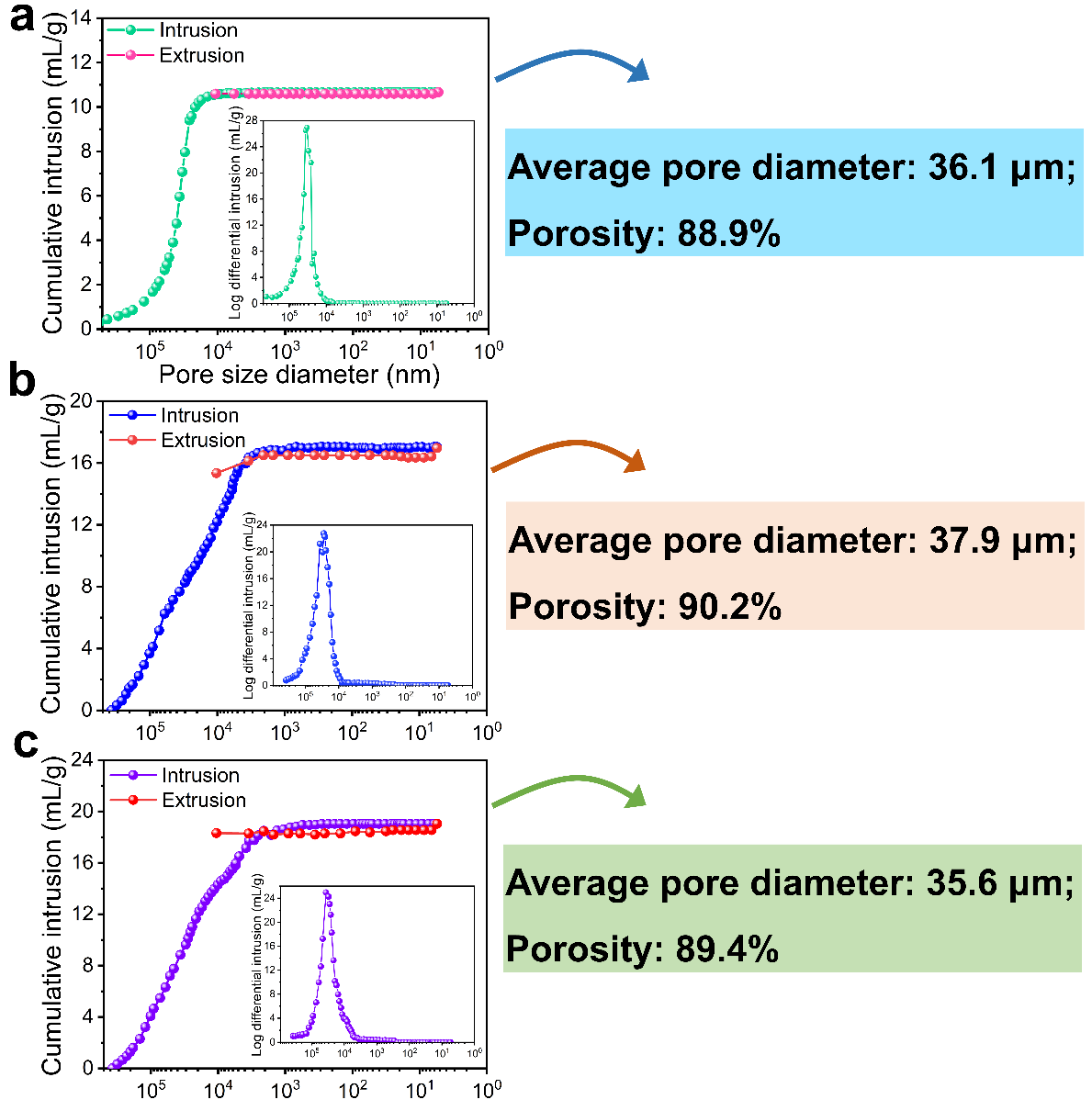


**Figure S21.** Representative pore size-distribution curves (inset) and corresponding average pore diameters of (a) ODA-based non-directional PI aerogel, (b) OT-46-based non-directional FPI aerogel, and (c) TFB-based non-directional FPI aerogel (upper layer) obtained from mercury intrusion experiments.


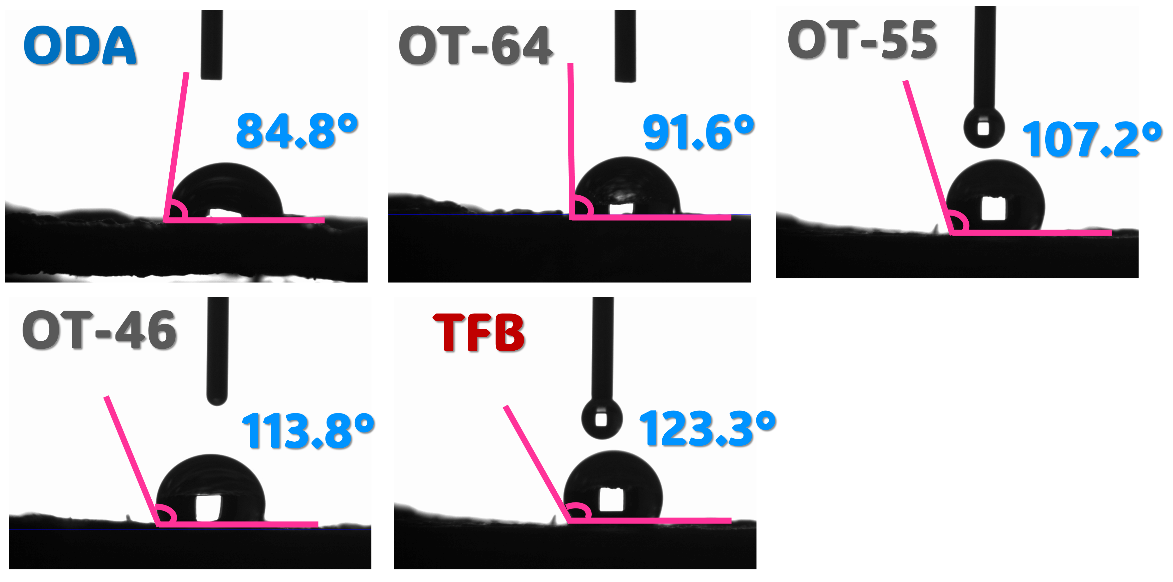


**Figure S22.** Water contact angle images of nondirectional FPI aerogels (upper layer).


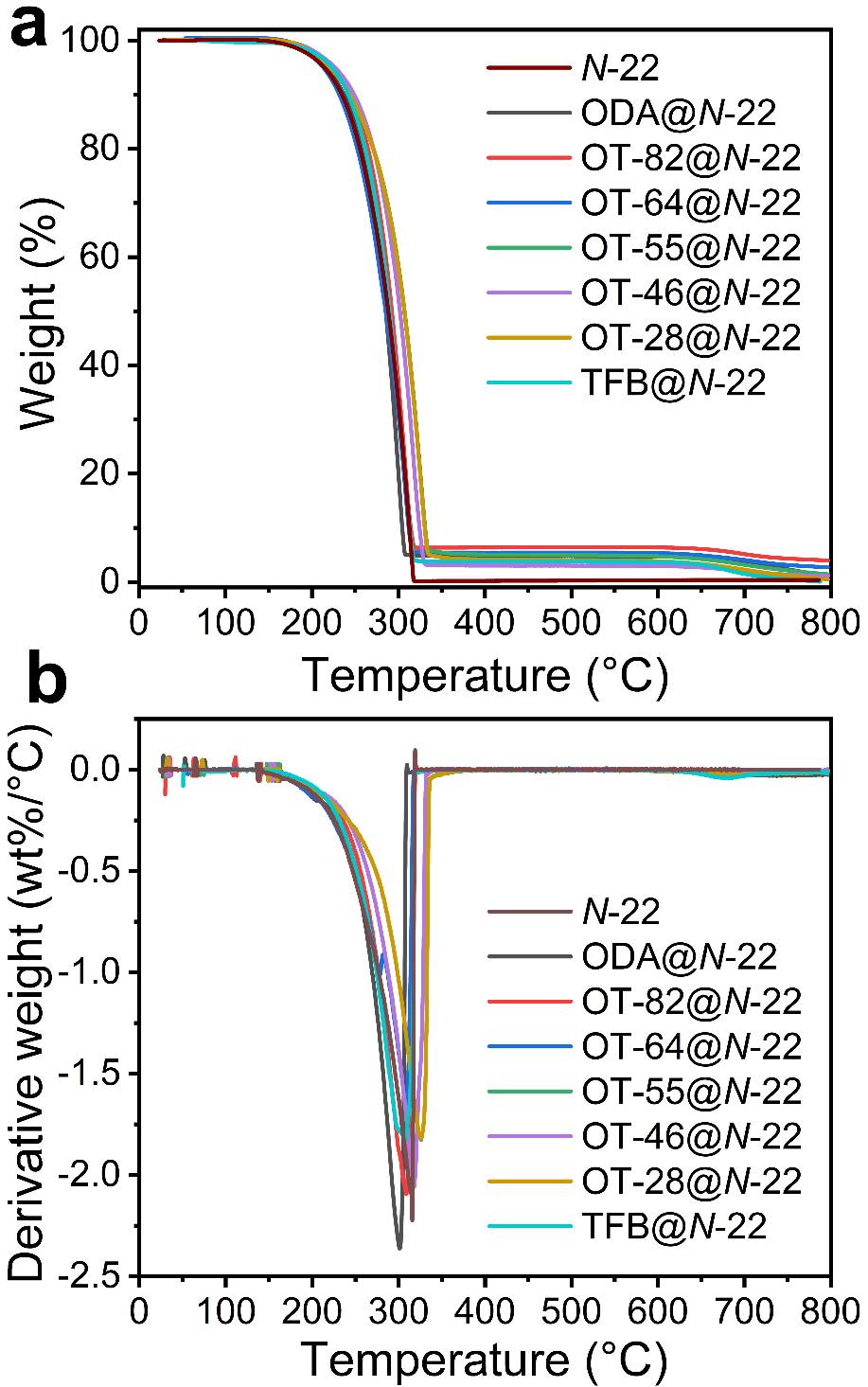


**Figure S23.** (a) TGA and (b) DTG thermograms of pure *N*-22 and nondirectional PI aerogel@*N*-22 composites with different contents of TFB.


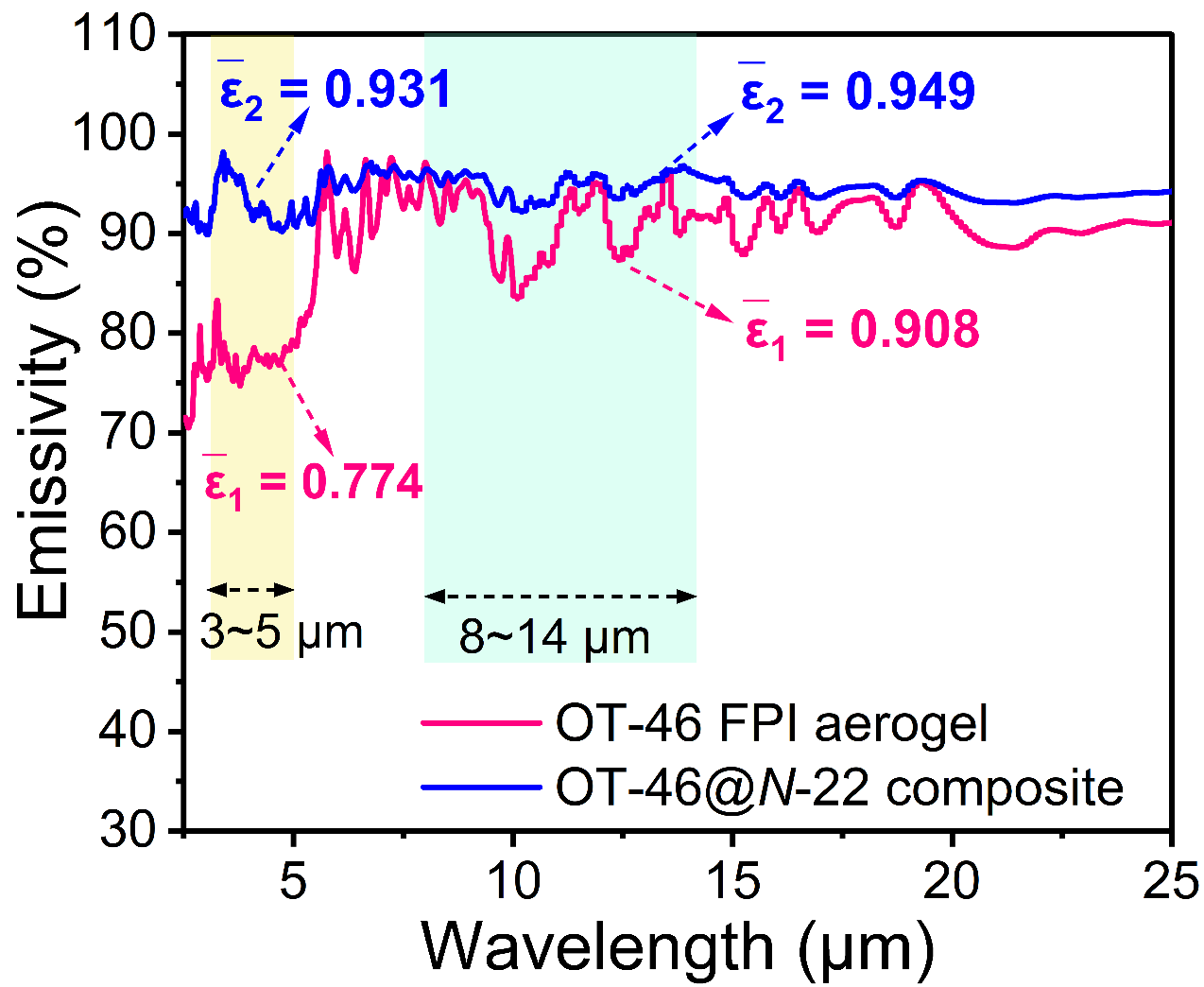


**Figure S24.** Thermal infrared emissivity spectrum of OT-46-based non-directional FPI aerogel and OT-46@*N*-22 composite (upper layer).


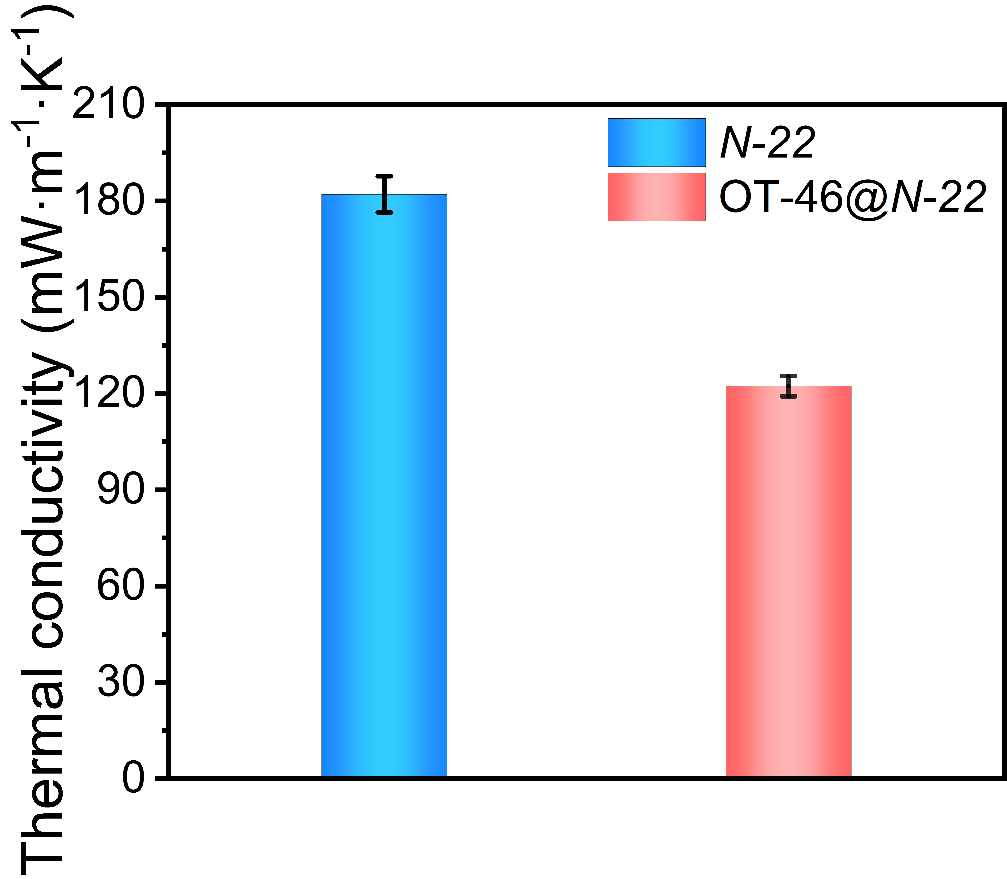


**Figure S25.** Thermal conductivities of pure *N*-22 and OT-46@*N*-22 composite.

~~
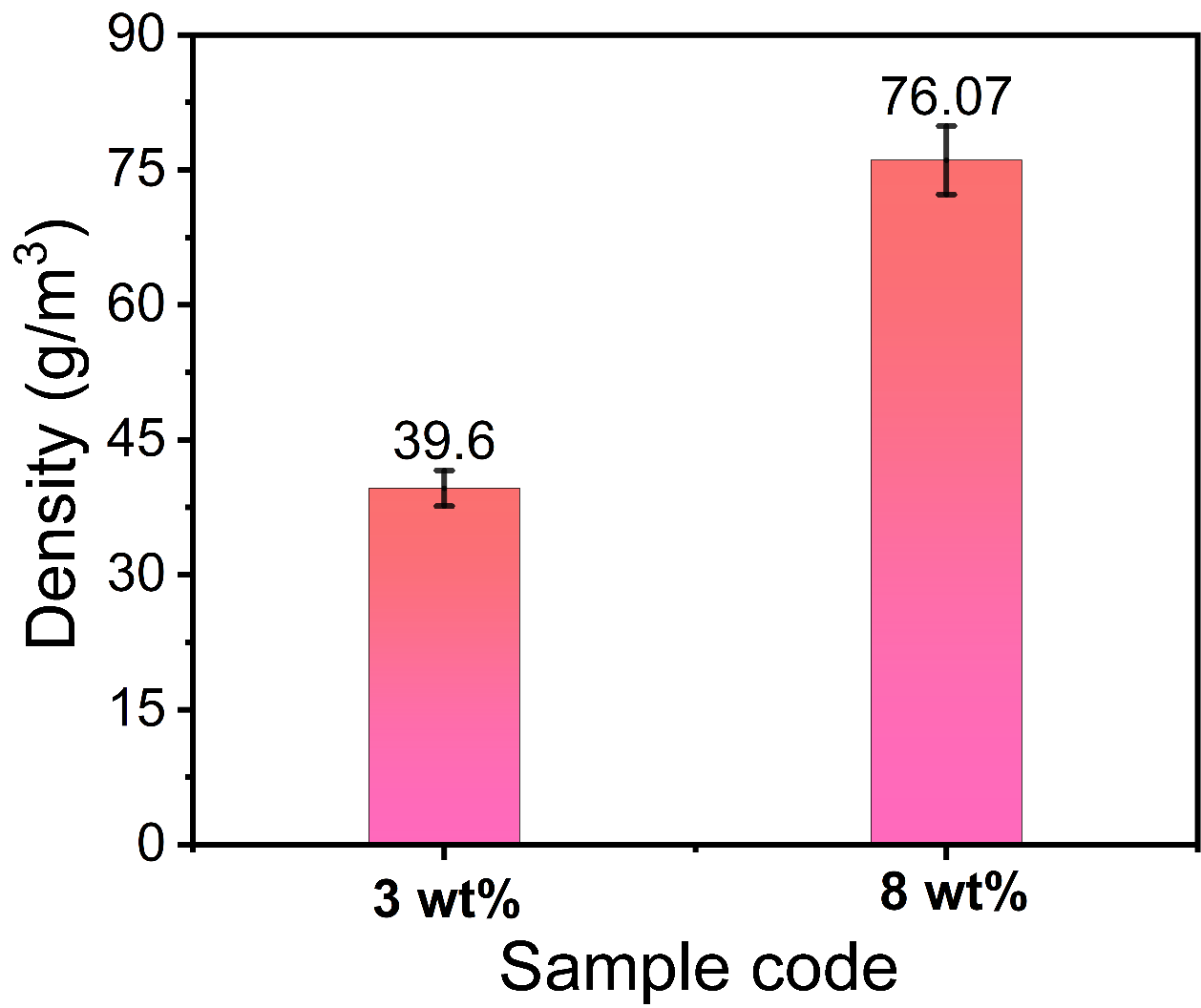
~~

**Figure S26.** Density of nondirectional PI aerogel synthesized at solid contents of 3 and 8 wt %.


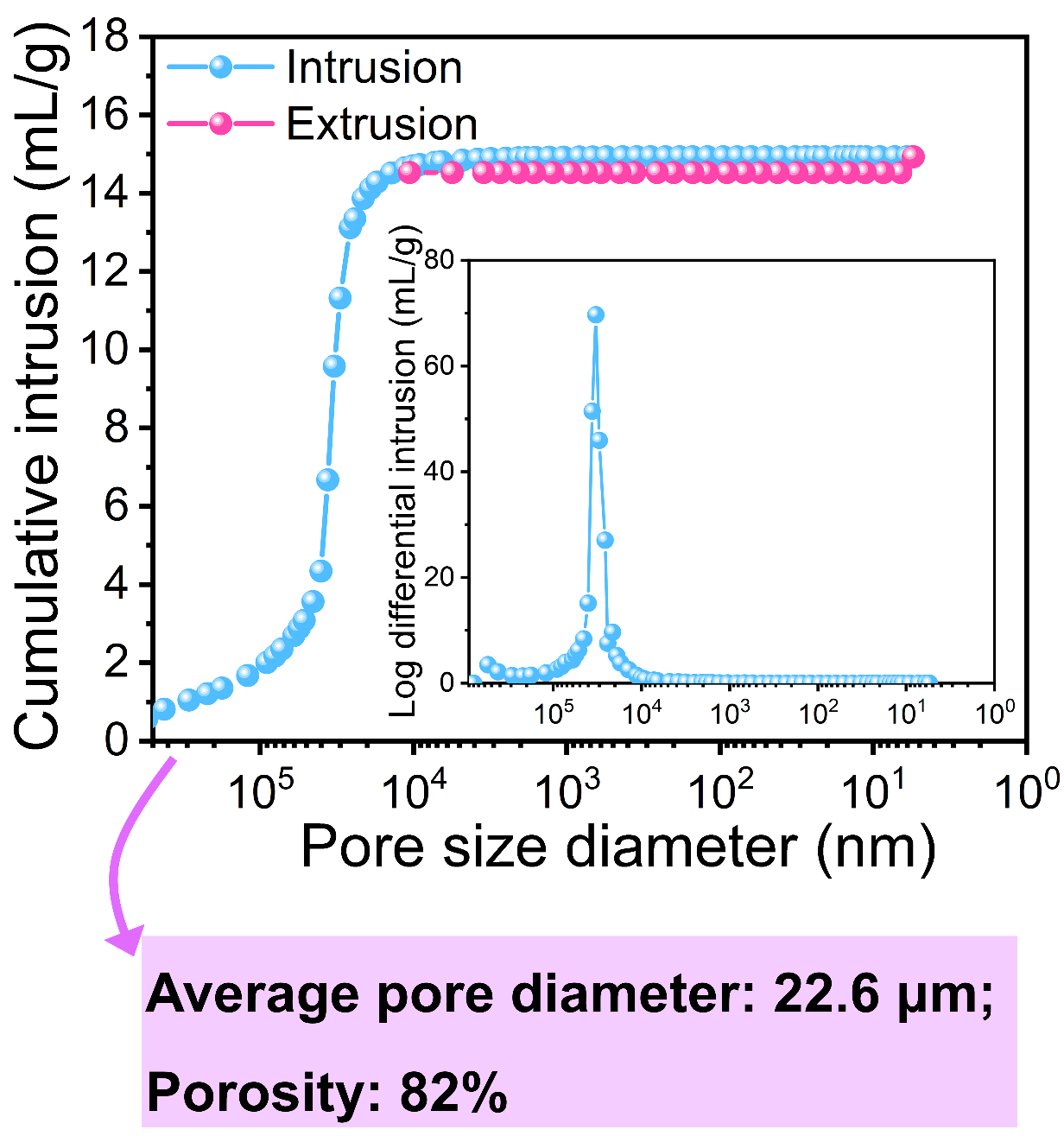


**Figure S27.** Mercury intrusion curves and pore size-distribution curves (inset) of ODA-based PI aerogel with a solid content of 8% (middle layer)

**
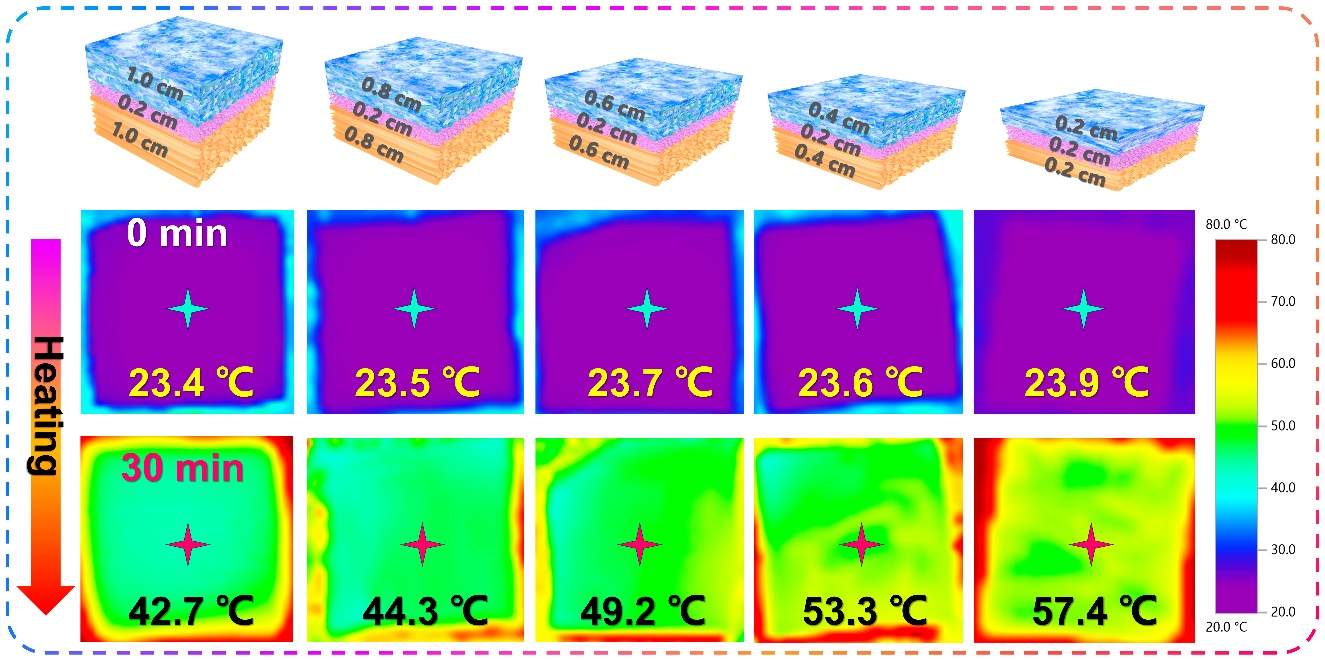
**

**Figure S28.** Infrared thermal images of sandwich-structured phase-change composites with different layer thicknesses during the heating process.


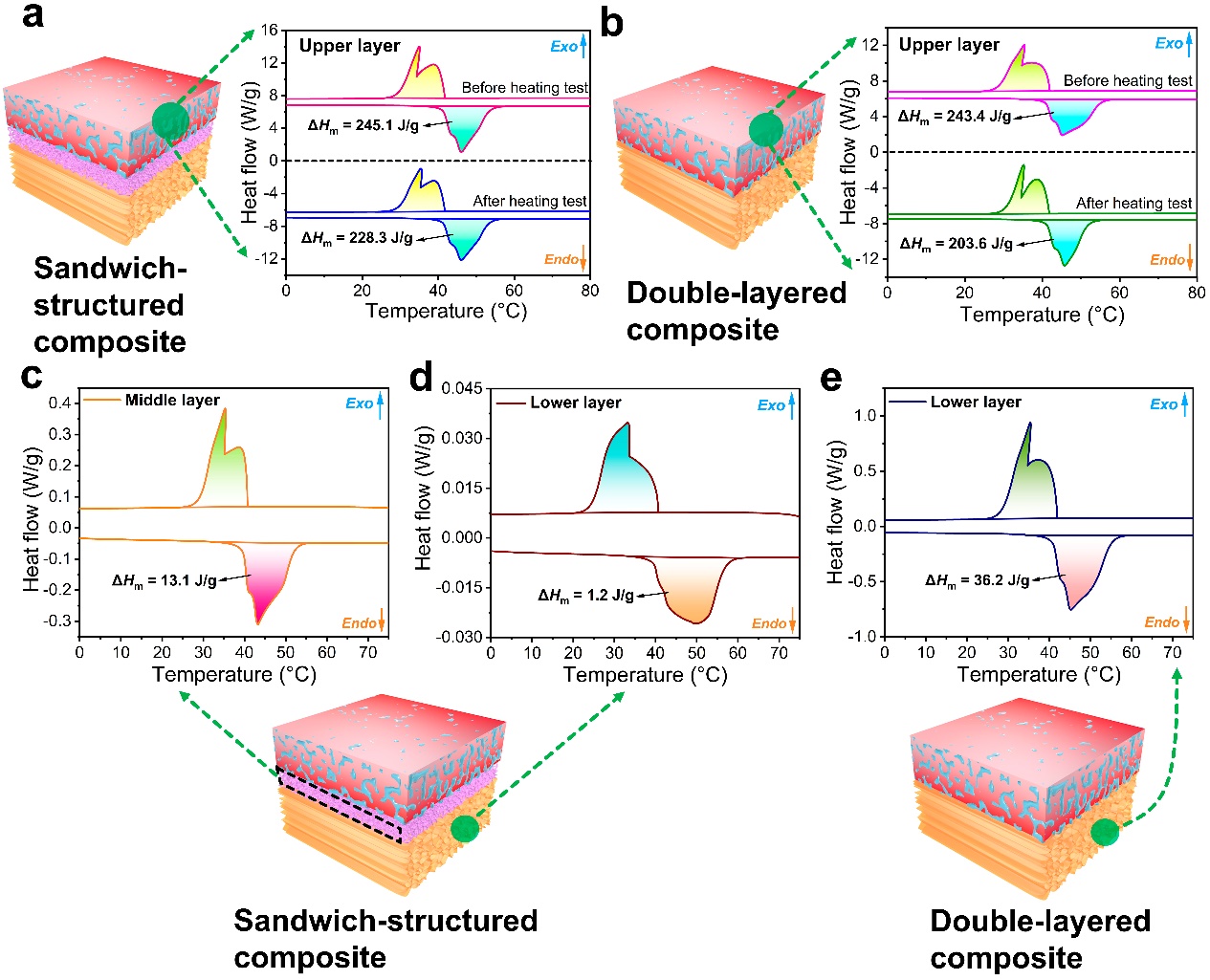


**Figure S29.** DSC thermograms of the upper layer of (a) sandwich-structured composite and (b) double-layered phase-change composite before and after heating at 100 ºC for 12 h. DSC thermograms of (c) the middle layer (d) lower layer of sandwich-structured composite after heating at 100 ºC for 12 h. (e) DSC thermograms of the lower layer of double-layered phase-change composite after heating at 100 ºC for 12 h.


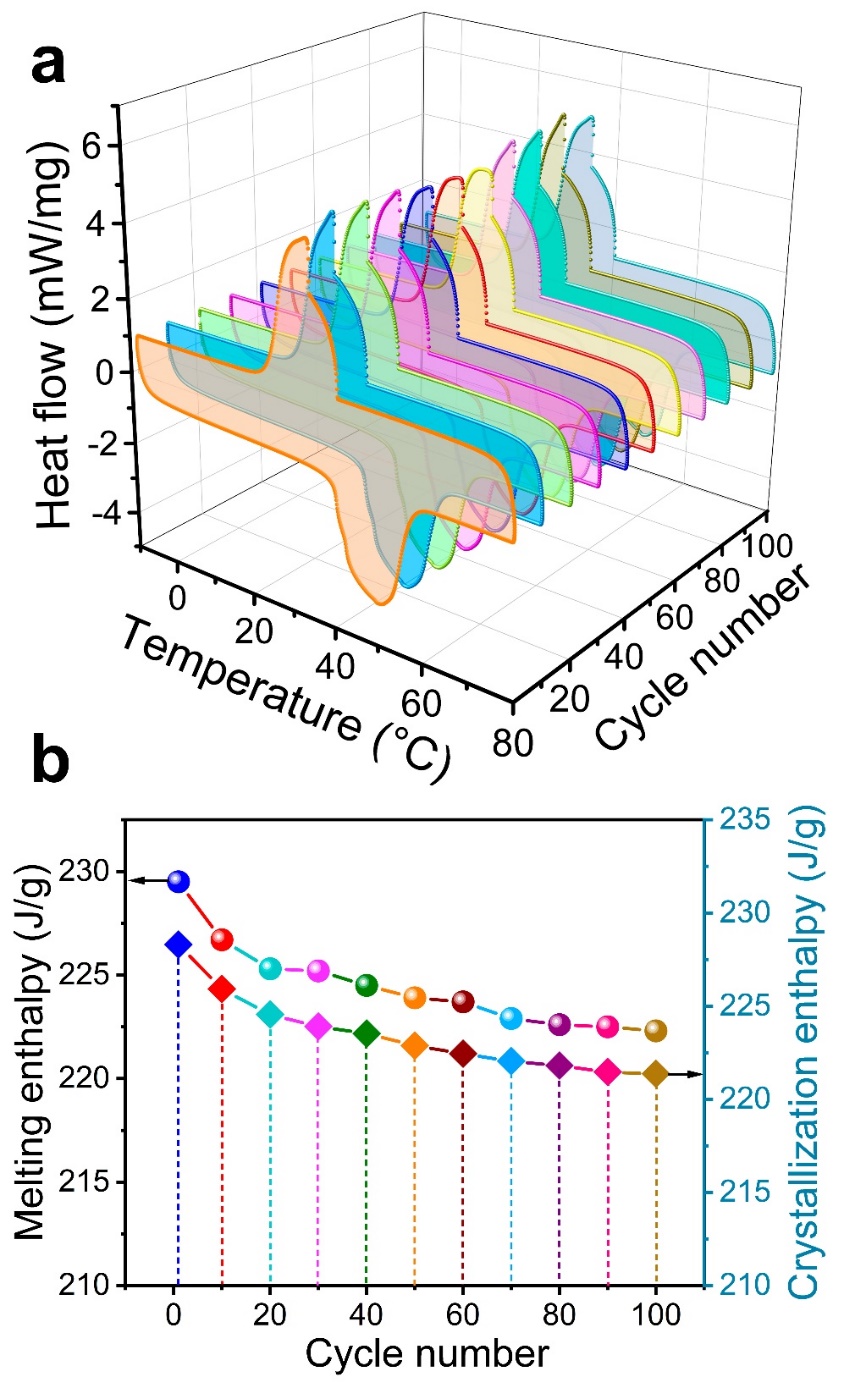


**Figure S30.** (a) DSC thermograms and (b) phase-change enthalpies of the nondirectional OT-46@*N*-22 composite as an upper layer at an interval of every 10 thermal cycles in the thermal cycling experiment.


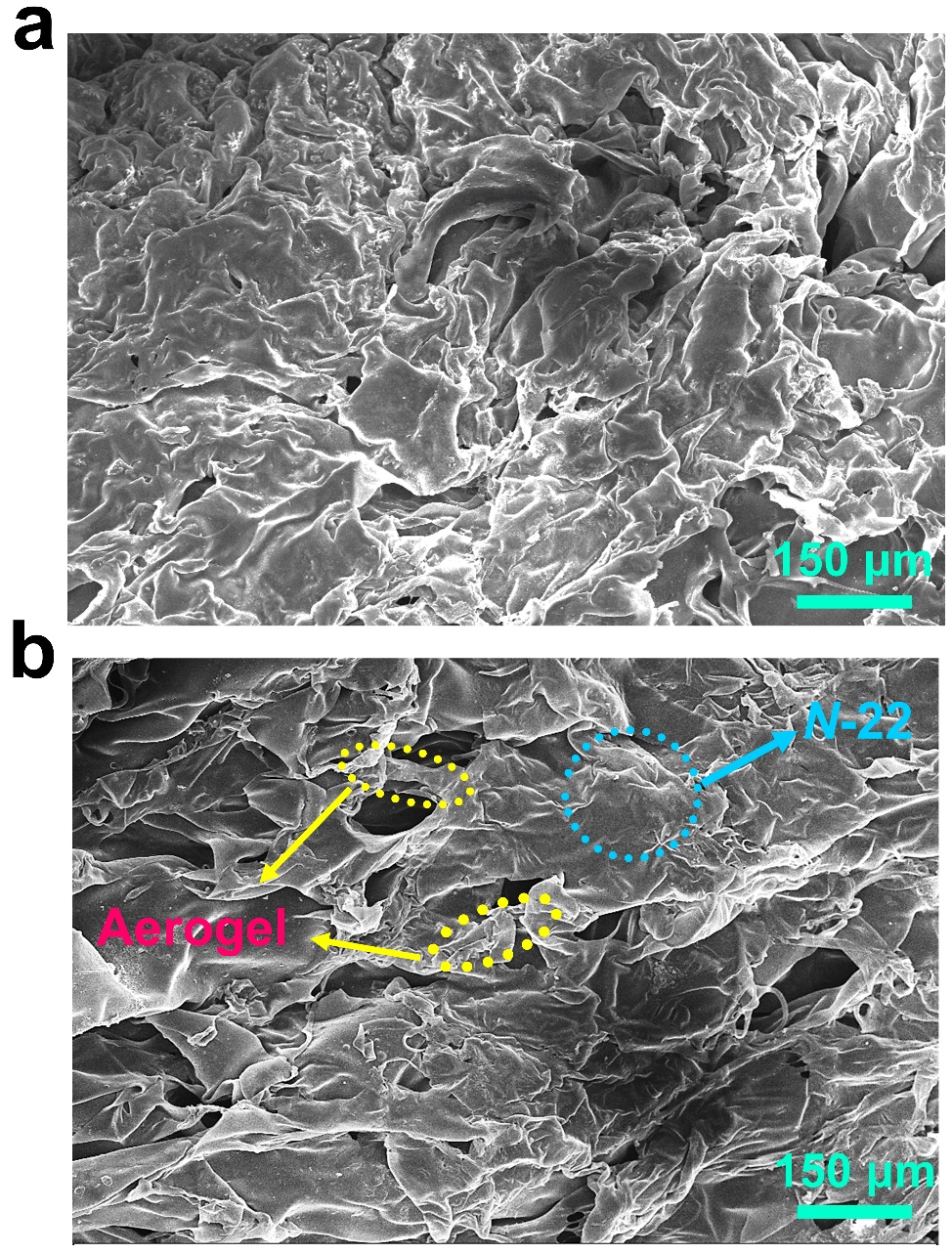


**Figure S31.** SEM images of nondirectional OT-46@*N*-22 composite (upper layer) after (a) the first and (b) 100th heating-cooling thermal cycles.


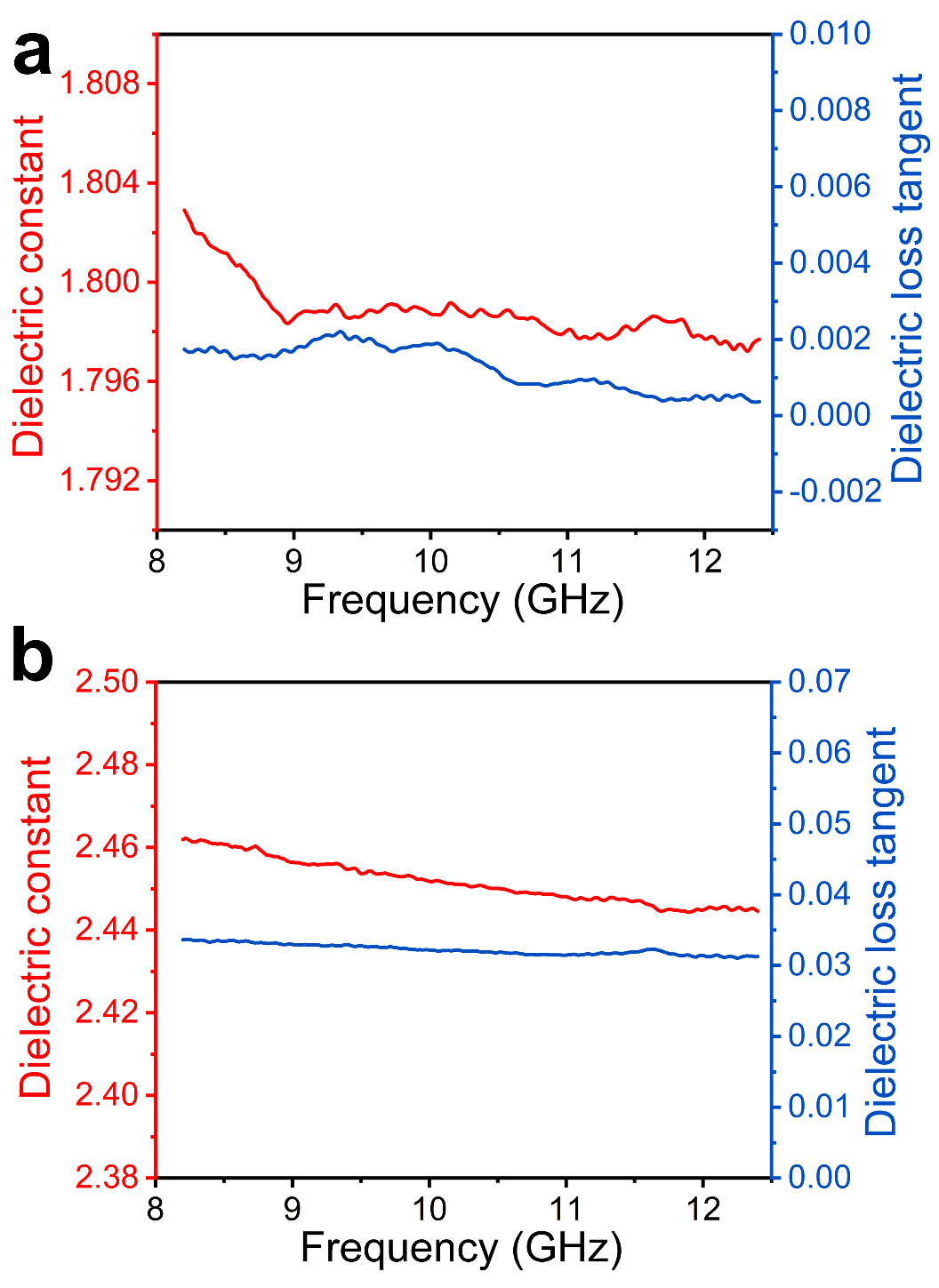


**Figure S32.** Dielectric constants of (a) upper layer and (b) middle layer of the sandwich-structured composite (sample dimensions: 22.9 × 10.2 × 2.5 mm^3^).

**Table S1.** The porous parameters and densities of unidirectional FPI aerogels with different contents of TFB.

| Sample | Average pore diameter (μm) | Specific surface area (m^2^/g) | Total immersion volume (mL/g) | Porosity  (%) | Density  (mg/cm^3^) |
| --- | --- | --- | --- | --- | --- |
| ODA-based PI aerogel | 30.9 | 7.6 | 13.0 | 86.0 | 43.9 |
| OT-82-based FPI aerogel | 34.6 | 4.6 | 13.3 | 92.1 | 35.4 |
| OT-64-based FPI aerogel | 32.4 | 5.4 | 16.7 | 90.0 | 34.1 |
| OT-55-based FPI aerogel | 38.3 | 4.3 | 19.2 | 93.3 | 34.5 |
| OT-46-based FPI aerogel | 37.1 | 8.1 | 14.2 | 93.5 | 32.9 |
| OT-28-based FPI aerogel | 32.8 | 8.4 | 19.8 | 87.9 | 38.7 |
| TFB-based FPI aerogel | 37.3 | 9.2 | 19.0 | 88.7 | 37.8 |

**Table S2.** The occupied volume, free volume, and fraction free volume of PI.

| Sample | Occupied volume  (Å^3^) | Free volume  (Å^3^) | Fraction free volume  (%) |
| --- | --- | --- | --- |
| ODA | 74704.36 | 44398.93 | 37.28 |
| OT-82 | 76775.09 | 45645.37 | 37.29 |
| OT-64 | 78908.83 | 47783.26 | 37.72 |
| OT-55 | 79927.41 | 48941.74 | 37.98 |
| OT-46 | 81055.15 | 51179.17 | 38.70 |
| OT-28 | 83114.16 | 51413.59 | 38.22 |
| TFB | 85239.73 | 55079.57 | 39.25 |

**Table S3.** The phase-change parameters of pure *N*-22 and *N*-22-based phase-change composites.

| Comosite sample | Crystallization enthalpy  (J g^−1^) | Melting enthalpy  (J g^−1^) | Crystallization temperature  (ºC) | Melting temperature  (ºC) |
| --- | --- | --- | --- | --- |
| Pure *N*-22 | 256.3 | 261.8 | 35.1 | 44.3 |
| ODA@*N*-22 | 233.8 | 238.1 | 32.9 | 45.1 |
| OT-82@*N*-22 | 236.0 | 237.4 | 29.7 | 50.7 |
| OT-64@*N*-22 | 220.1 | 224.3 | 33.9 | 43.4 |
| OT-55@*N*-22 | 226.1 | 233.6 | 32.9 | 49.5 |
| OT-46@*N*-22 | 237.8 | 242.7 | 35.3 | 43.2 |
| OT-28@*N*-22 | 225.7 | 233.9 | 31.6 | 51.4 |
| TFB@N-22 | 226.4 | 226.9 | 30.4 | 51.5 |

**Table S4.** Values of the relevant parameters for the calculation of the ratio of open pores of the ODA-based PI aerogel with a solid content of 8 % (middle layer).

| Parameter | Value |
| --- | --- |
| Experimental density of PI aerogel (*ρ*_1_) | 0.076 g cm^−1^ |
| Theoretical density of PI (*ρ*_2_) | 1.38 g cm^−1^ |
| Porosity of PI aerogel calculated by mass and volume (*P*_1_) | 94.5% |
| Porosity of aerogel obtained by mercury intrusion (*P*_2_) | 82% |
| Ratio of open pores (%) | 86.8% |

The ratio of open pores can be calculated by the following equations.

$$\begin{matrix} P_{1}=(1-\frac{\rho_{1}}{\rho_{2}})\times100\% \\ Ratio\text{ of open pores}=\frac{P_{2}}{P_{1}}\times100\% \end{matrix}$$
